# Building bridges to emotion: Developing a standardized film-based emotion elicitation tool for Iranian culture

**DOI:** 10.1371/journal.pone.0343598

**Published:** 2026-03-12

**Authors:** Milad Yousefi, Jamal Amani Rad

**Affiliations:** 1 Institute for Cognitive and Brain Sciences, Shahid Beheshti University, Tehran, Iran; 2 Choice Modelling Centre, University of Leeds, Leeds, United Kingdom; Nanyang Technological University, SINGAPORE

## Abstract

Emotion elicitation through culturally relevant stimuli is crucial for psychological research that seeks to explore affective processes within specific populations. This study aimed to develop and validate a film-based emotion elicitation tool for Iranian culture. A comprehensive database of short video clips was selected to evoke distinct emotional states, including happiness, tenderness, fear, anger, sadness, and disgust, alongside neutral clips as controls. To validate the database, the emotional responses of 300 Iranian participants were assessed using key dimensions of arousal and valence, positive and negative affective states, gender differences, and mixed emotions. The results indicated that all emotional stimuli elicited significantly higher arousal levels compared to neutral clips, with fear-inducing clips generating the highest arousal levels. In terms of valence, positive emotional films, such as those inducing happiness and tenderness, were significantly associated with higher pleasantness, while anger elicited the lowest valence scores, indicating its strong negative impact. Additionally, the video clips effectively differentiated between positive and negative affective states, with clear statistical significance observed across all comparisons (p<0.0001), showing that videos designed to evoke positive emotions (e.g., happiness) and negative emotions (e.g., fear) successfully achieved these outcomes across the participant group. Gender differences were also examined, with women generally showing higher levels of emotional arousal than men, particularly in response to happiness, tenderness, sadness, and disgust, though the overall effect sizes were small. Finally, the study delved into the complexity of mixed emotions, where participants often experienced simultaneous conflicting emotions, such as happiness and sadness, challenging traditional discrete emotion frameworks. The findings affirm the cultural relevance and efficacy of the developed video clip database in eliciting a wide range of emotional responses, making it a valuable tool for future psychological studies in Iranian contexts. This study underscores the importance of culturally specific stimuli in emotion research and provides a robust resource for exploring the emotional landscape within Iranian culture.

## Introduction

Over the past several decades, the field of emotion research has experienced a significant surge, particularly in its exploration of the intricate relationships between emotion, cognition, behavior, personality, and physiology. The importance of employing emotion-regulatory strategies for various cognitive processes is being recognized significantly. Such recognition has resulted in higher dependency on the laboratory paradigms that use emotional elicitation stimuli to modify, mimic, or induce emotional contexts for comprehensive research. Such a methodology is used extensively across the social sciences with a specific concentration on psychology, for investigation of different fields, e.g., emotional mimicry and contagion, mood dynamics, socio-cultural and intrapersonal processes, emotional regulation, and prosocial behavior [1–6]. To artificially induce emotional changes for these studies, a variety of techniques have been developed, including the use of emotional words [7,8], English texts [9–12], emotional images [13–17], faces [18–20], video clips [21–37], music [38–42], personal recollection [39,43–46], imagination [47–50], and virtual reality [51,52].

Despite the efficacy of all these methods in eliciting discrete/continuous mood states, there is a growing trend towards the use of emotional audiovisual materials or film clips. This trend reflects their effectiveness in eliciting the desired subjective emotional states for research purposes, making them one of the most user-friendly techniques in the laboratory setting. These clips, serving as an optimal artificial model of reality, are dynamic, multi-modal, and replete with contextual information that facilitates the understanding of characters’ emotional states [29,53–55]. Their complexity, coupled with a blend of explicit and implicit affective and cognitive features, effectively engages the audience’s auditory and visual senses [56–58], leading to an emotional experience akin to real life, where emotions evolve over time [53,59]. This method effectively addresses potential ethical and practical concerns related to the manipulation of emotions [60]. Moreover, given the ubiquity of television and film viewing and the global target audience of most contemporary films, it is assumed that the video presentation induction procedure is not likely to be largely influenced by gender and other differences. Consequently, film clips provide a more ecologically valid alternative for emotion elicitation compared to other affect-induction techniques, such as static images and recalling previous emotional events [56,61–64]. Furthermore, emotional film clips have demonstrated their capacity to induce robust and quantifiable subjective and physiological changes [64–66]. Finally, meta-analyses of emotion induction further underscore the potency of film as one of the most effective ways of eliciting emotions [55,67–69]. Together, these studies furnish some databases of film clips that are expected to consistently evoke specific responses from participants. However, the validity and relevance of the normative data associated with some clips are being re-evaluated due to societal shifts in preferences and norms over the past two decades (e.g., [70]), highlighting the need for a regularly updated archive for research utilization.

Recognizing the profound influence of personal, gender-specific, cultural, and linguistic factors on emotions (e.g., [53,71–76]), particularly complex emotions (e.g., [31,32,77–79]), it becomes imperative to validate emotional assessment tools across diverse cultures [80,81]. In other words, despite the development of a robust and reliable collection of video clips over several decades, their validity in different cultural contexts remains uncertain due to the aforementioned factors. Hence, it is both theoretically and practically significant to investigate the effectiveness of these video clips in evoking corresponding emotional responses in diverse cultures. This study aims to fill a significant gap in the literature by validating a database within the Iranian community, thereby addressing a wide spectrum of potential research inquiries – an endeavor yet to be accomplished. This initiative has led to the development of culture-sensitive emotional databases catering to non-English speaking audiences, a feat already achieved in other cultures and languages. For instance, Schaefer et al. [23] developed a database for French-speaking audiences, encapsulating emotions such as amusement, tenderness, anger, contentment, fear, sadness, and neutrality. Similarly, Xu [82] introduced several Chinese music videos expressing happiness, anger, contentment, fear, sadness, surprise, and neutrality. Hewig et al. [63] not only validated previous movie sets but also enriched the German sample with four neutral clips, encompassing emotions like happiness, amusement, contentment, fear, sadness, anger, and neutrality. This process of validation has been investigated in other cultures and languages as well, with further examples available in [24,60,83–87]. Importantly, the Iranian specificity of the present database extends beyond language adaptation; candidate excerpts were screened against an explicit cultural/religious compatibility criterion applied to both visual content and dialogue, prior to final inclusion. This study, therefore, stands as a pioneering effort in expanding the horizons of emotional research within the Iranian community. Consequently, we have developed and evaluated of what is to the best of our knowledge the most comprehensive normative database of emotional film scenes within Iranian culture. This paper intends to detail the development of this database, including the tests that demonstrated its efficacy in inducing emotions in a laboratory environment.

The creation and validation of a database for video clips that elicit emotions should be firmly rooted in distinct theories of emotion. A vibrant theoretical debate has emerged concerning the intrinsic nature of human emotional responses. Theorists of discrete or basic emotion theories, such as Ekman [88,89] and Tooby & Cosmides [90], argue that emotions are short-lived, organized into a limited set of fundamental categories, and consist of distinct episodes that include various loosely connected responses—autonomic, behavioral, and experiential—that have evolved to facilitate our adaptation to specific environmental challenges. For instance, anger involves a set of responses developed to address goal obstruction [91], while sadness involves responses tailored to cope with loss [92]. These viewpoints have facilitated the development of sets of emotional film stimuli capable of eliciting distinct experiential states corresponding to basic emotions (e.g., happiness, surprise, fear, sadness, anger, and disgust). However, beyond emotional discreteness, research often requires stimuli validated on broader criteria, including films that assess the impact of varied emotional arousal and valence intensities on cognitive functions. Furthermore, the elicitation of mixed emotions – where multiple basic emotions are experienced simultaneously – is crucial for comprehensive studies [93–95]. Dimensional or psychological constructionist theorists, such as Barrett [96], propose that emotional responses are more accurately understood through two underlying neurobiological continuous dimensions [97–99] that respond to environmental stimuli, rather than through discrete basic emotions: hedonic valence (i.e., pleasantness or unpleasantness) and arousal level (i.e., low or high) of stimuli that shape our multi-dimensional responses (behavioral, autonomic, etc.). Hence, specific emotions such as anger, fear, or sadness are perceived as social constructs that originate from how individuals appraise or conceptualize the provoking event [100].

Thus, to construct a comprehensive collection of emotional stimuli, researchers should manipulate both these dimensions as well as basic emotions. This supports the selection and arrangement of a diverse assortment of film clips, which are instrumental for researchers with varying theoretical orientations or research objectives. For instance, researchers aiming to manipulate mood to study its impact on executive attention may find clips validated within a dimensional framework particularly useful. In contrast, those investigating the effects of fear on visual search and memory might prefer clips validated under a discrete emotions framework. Accordingly, we have examined the movie clip stimuli through a dual-dimensional lens. The first dimension involves the analysis of stimuli known to evoke specific or discrete emotional reactions, while the second dimension pertains to stimuli that align with dimensional or psychological constructionist perspectives. Moreover, our analysis categorizes stimuli that predominantly utilize a discrete emotions framework, yet acknowledges the intricacies involved in the simultaneous elicitation of related emotions.

### Overview

As indicated, culture and language are pivotal elements that significantly influence the effectiveness of emotion induction techniques. Over recent decades, there has been a substantial upsurge in the attention given by Iranian researchers to the study of emotions and their profound impact on various facets of human behavior and its underlying processes, including individual, social, cognitive, and neural functions. This has led to a multitude of studies in this domain (e.g., see [101–105]). However, it is noteworthy that mood induction methods such as images [106, 107, 108], words [109,110], and music [111–114] have been predominantly used, overshadowing the use of video clips. Although a variety of video clips have been utilized in a handful of studies, their use has been rather limited and confined to specific research contexts. Notably, recent work by [26] has contributed to this area by not only collecting self-report data but also integrating neuropsychological approaches and collecting various neural data. However, our study differs significantly in several key aspects. We assessed a substantially larger sample size of 300 participants, which enhances the statistical power and validity of our findings. Additionally, we have strived to capture the unique cultural diversity of Iran, a multicultural society comprising Persian, Kurdish, Turkish, and other ethnic groups, to ensure a more representative and inclusive dataset. Furthermore, while [26] focused on locally Iranian-produced films, our study adopts a different approach by utilizing internationally recognized films with validated Persian subtitles, following methodologies similar to those used in cross-cultural emotion studies (e.g., [23]). This approach is based on the hypothesis that basic emotions are likely to be consistent across cultures, thus enabling a more reliable induction of emotions within the Iranian context. The specific differences and advantages of our approach, including the inclusion of the emotion “tenderness,” mixed emotions, gender differences, and the validation of the Discrete Emotions Scale (DES) for the first time in the Iranian community, will be further discussed in the Discussion section.

With this in mind, the primary objective of the present study is to develop and validate a reliable and comprehensive database of emotion-inducing video clips as a methodological tool for emotion research within the Iranian culture and community. This objective encompasses the following goals: 1. Selection from a fairly large collection of diverse video clips that span a broad spectrum of emotional dimensions. 2. Evaluation of the effectiveness of video clips utilizing various tools from both dimensional and discrete approaches. 3. Creation and validation of a video clip database encompassing seven emotions: neutral, sadness, happiness, fear, anger, tenderness, and disgust. 4. Identification of the most emotionally effective video clips. 5. Translation and validation of a discrete emotion questionnaire for the first time within the Iranian community. 6. Lastly, providing open access to the data, analyses, and codes via the Open Science Framework, facilitates flexible selection and usage by researchers worldwide.

The remainder of this paper is structured as follows: We first delve into the ’Methods’ section, where we illuminate the methodologies that anchor the foundation of this study. This includes an exhaustive explanation of the development of our video clip database, detailing the process of clip selection and collection, the method of use in a laboratory setting, and a thorough description of the process to evaluate the database’s effectiveness as emotional stimuli. This involves the use of tools to assess emotions in both discrete and continuous dimensions. The discrete dimension employs an extended version of the Differential Emotional Scale [23,115–117], validated for the first time in Iranian culture in this paper, which aligns with a basic emotions approach. The continuous dimension uses the Self-Assessment Manikin [102,118,119], validated by Nabizadeh et al. [120] for the Iranian community, to evaluate the video clip database aligned with a dimensional approach to emotions – subjective arousal and pleasantness. This section also covers the experimental design, participant involvement, and the analytical procedures used. Next, we present the ’Results’ section, where we reveal the findings of our data analysis, addressing a wide array of research questions. This provides insights into the process of selecting and ranking the most effective video clips from the database, supplemented by results from the highest-scoring subgroups. This leads into the ’Discussion’ section, where we undertake a meticulous analysis of our findings. Finally, we conclude with the ’Conclusion and Future Works’ section, summarizing the key findings of the research and suggesting potential directions for future exploration.

## Methods

### Preliminary review of films and final selection by experts

The first step involved the careful compilation and examination of a diverse set of film excerpts, each corresponding to one of seven distinct emotional categories. These categories were chosen in alignment with the principles of the “basic emotion theory” and their established use in prior studies. The emotions under investigation in this study included neutral, sadness, happiness, fear, anger, tenderness, and disgust. The selection of these video excerpts was governed by a set of five stringent criteria:

- The visual and verbal components of the films should align with the cultural and religious structures of Iranian society.
- The films often fall into the category of emotions delineated in the “basic emotion theory.”
- The verbal cues used in the films should be easily comprehensible and commonly found in everyday language.
- A majority of the selected films should have been utilized in prior emotion-related research.
- The elicited emotion should remain consistent throughout the duration of the film, thereby excluding films that evoke a mix of positive and negative emotions.
- The film clips should be relatively brief, devoid of visible watermarks, logos, or mosaics, and should not be of the animated genre.

Although tenderness is not typically categorized as a basic emotion, its inclusion in this study was justified due to its acknowledgment as a distinct category of positive attachment-related emotions in prior studies [23,121–123]. Furthermore, this emotion is effectively elicited by films. We therefore included tenderness to complement happiness with a conceptually distinct positive category that has also been considered in prior film-clip databases (e.g., [23]). By contrast, we did not designate surprise as a target category because it is often brief and valence-ambiguous and can rapidly transition into other emotions, making sustained induction within multi-second excerpts less compatible with our selection criterion that the elicited emotion should remain relatively consistent throughout a clip; nevertheless, surprise-related experience was still assessed via the DES item cluster (“surprised, amazed, astonished”).

In our study, we drew upon prior research on the development and validation of mood-induction films to compile an extensive collection of film excerpts. Initially, we extracted 130 movie excerpts from a vast pool of potential scenes from previous studies. Subsequently, a collaborative survey involving the study’s researchers and six research assistants (including four females and four males) led to the selection of four video clips for each emotional category. The final selection included films that were commonly chosen by the individuals involved. Our selection methodology was subjective, aligning with the approach adopted in previous studies [22,29,86]. The research assistants were instructed to select movie excerpts that could potentially induce specific emotions, including neutrality, sadness, happiness, fear, anger, tenderness, and disgust. To ensure a clear understanding of these subjective emotions, eight research assistants were trained in various emotional categories, drawing upon the theoretical psychological knowledge presented in the previous section. Their training was validated by assessing their understanding of several common materials from current databases. In total, 27 selected video clips were incorporated into the study, with four movies representing each emotion, except for the neutral emotion, which was represented by three finalized movies. However, the research group unanimously agreed that the movies associated with the “happiness” emotion from previous studies were not suitable for inducing the desired mood within the Iranian community. Consequently, four “happiness” movies were selected and introduced into the study for the first time by the researchers. The finalized video clips ranged in length from 16 to 354 seconds, averaging 162.67 s (SD = 97.80 s). All movies were in English, with Persian subtitles added. These subtitles were meticulously reviewed and approved by three cognitive linguistics experts. We opted for subtitles over dubbed versions for two reasons: firstly, to preserve the original texture of the movies, particularly the soundtrack, which plays a crucial role in mood induction; and secondly, because in Iranian society, watching foreign movies with subtitles is more prevalent than watching dubbed versions. The details of each emotional film are shown in Table 1.

**Table 1:** Description of the video clips.

Title (clip name)	Length (s)	Target emotion	Induced affect	Clip description	References
Life is Beautiful 48	227	Tenderness	Positive	Within the stark confines of an internment facility, a father deftly crafts a misleading interpretation of an officer‘s utterances, solely to insulate his child from the pervasive dread.	[23,68,128]
The Champ 1979	170	Sadness	Negative	Son’s last embrace with his dying champion father.	[22,23,63,68]
Road Kill	302	Fear	Negative	An assassin cunningly entraps two unsuspecting boys in a hotel chamber, where opening the door triggers a shotgun, menacingly aimed at their comrade.	[29]
The Dead Poets Society	160	Tenderness	Positive	In the climactic scene of “The Dead Poets Society,” students ascend their desks in a silent yet potent act of solidarity, honoring Mr. Keating as he faces his unjust dismissal.	[23,68]
The Piano	45	Anger	Negative	A character in ‘The Piano‘ grimly loses a finger.	[23,68]
American History X	80	Anger	Negative	In ‘American History X‘, a neo-Nazi commits a heinous act against an African-American man, forcefully crushing his head against the curb.	[23,68]
Hellraiser	90	Disgust	Negative	Two ominous stains on the floor begin to expand. Gradually, they morph into a grotesque creature that eerily mirrors the structure of a human skeleton.	[23,68]
Child	67	Fear	Negative	Chucky mercilessly assaults Miss Kettlewell with a ruler, delivering fatal blows to her abdomen.	[23]
Lost	137	Sadness	Negative	Two characters drown together in a poignant scene.	[23]
Blue 1	16	Neutral	Neutral	Car wheels spin; a hand meets the open wind.	[23,54]
Mr. Bean Exam	201	Happiness	Positive	Mr. Bean attempts to navigate an exam with unconventional and hilariously unsuccessful cheating methods.	
A Perfect World	279	Sadness	Negative	The character Butch Haynes meets a tragic end as law enforcement officers fatally shot him following a tense pursuit and standoff.	[23]
The Silence of the Lambs	218	Disgust	Negative	Investigators conduct a detailed forensic examination of a deceased victim.	[22,23]
The Dentist	56	Disgust	Negative	A man shockingly discovers a severed tongue in the pool.	[23,54]
Life is Beautiful 70	248	Tenderness	Positive	Mother and son experience a reunion.	[23,128]
Blue 2	25	Neutral	Neutral	Emerging from the subway, someone steps into the market.	[23,68]
Blue 3	43	Neutral	Neutral	Girl enter the car, pass the market, and reach the house.	[23,68]
Charlee Prison	330	Happiness	Positive	Chaplin’s character, mistaking a substance for salt in the prison canteen, consumes his meal and becomes extremely energetic. This leads to him inadvertently preventing a prison break, earning early release.	
Once Were Wa	128	Anger	Negative	A domestic dispute escalates into a brutal assault when the wife refuses to cook for her husband’s friend.	[29]
The Professional	354	Anger	Negative	In Léon: The Professional (1994), while Mathilda is at the supermarket, her family is brutally gunned down at home, with her brother hiding under the bed.	[23]
Indiana Jones	117	Disgust	Negative	In a dark cave, characters encounter numerous skulls and an unsettling swarm of black rats.	[23]
Charlee Box	260	Happiness	Positive	Chaplin’s character unexpectedly finds himself in a boxing match.	
Copycat	147	Fear	Negative	A young girl’s struggle to accept her friend’s death is depicted at his funeral.	[23]
My Girl	135	Sadness	Negative	A young girl’s struggle to accept her friend’s death is depicted at his funeral.	[29]
Charlee Lion	206	Happiness	Positive	Chaplin’s character finds himself inadvertently trapped within a lion’s cage	
Life is Beautiful 44	103	Tenderness	Positive	Within the stark boundaries of a detention facility, a father and his son communicate with the mother, their voices amplified by a loudspeaker, resonating throughout the entire camp.	[23,128]
The Shining	266	Fear	Negative	A man chillingly descends into madness, culminating in a scene where he pursues his wife, wielding an axe with deadly intent.	[22,23,63,68]

### Participants and ethics

A diverse cohort of three hundred native Iranians, comprising an equal distribution of males and females within the age range of 18 and 30 years (mean age = 24.44, SD = 3.66), voluntarily participated in the experiment through face-to-face sessions conducted at a dedicated behavioral laboratory. Comprehensive metadata, encompassing age, gender, and other demographic details of the participants, are readily accessible in the project’s open-source framework. All participants had either normal or corrected-to-normal visual and auditory functions. Prior to their participation, they were provided with exhaustive briefings pertaining to the research objectives and the experimental procedures. Following these briefings, they provided their consent to participate in the study by signing the requisite forms. The participants were assured of the confidentiality of their responses and were informed that they could withdraw from the study at any time. All participants completed the full experimental session and provided complete responses. Thus, no participants were excluded after enrollment and no data were discarded due to missing or incomplete responses. The task was programmed such that participants could proceed only after completing the required ratings, ensuring complete response records for all participants. This study received ethical approval from the Research Ethics Committees of Shahid Beheshti University (Approval ID: IR.SBU.REC.1399.066, Date: 2020-01-10). No participants under the age of 18 were included. Participant recruitment occurred between May 15, 2021 and August 20, 2021. Data collection (i.e., in-lab testing sessions) was completed within this period, and the full sample of 300 participants was tested over approximately 14 weeks, with sessions scheduled continuously across the recruitment window until completion. Demographic metadata (e.g., age and gender) are accessible via the project’s open-source framework. All data and programming codes supporting the findings of this study are available on the Open Science Framework (https://osf.io/2td5n/).

In our study, participants were carefully screened to ensure eligibility. Specifically, they were not receiving psychotropic treatment or using drugs, and they reported no history of psychological, psychiatric, or neurological disorders, consistent with DSM-5 criteria. To minimize potential bias due to depressive symptomatology in affective responding, participants completed the Beck Depression Inventory-II (BDI-II) [124] prior to the experiment. The BDI-II is a widely used self-report instrument assessing depressive symptoms over the past two weeks; following commonly used thresholds [125], individuals scoring ≥16 were not enrolled in the study. The BDI-II has well-established psychometric properties [126] and has also been validated in Iranian samples, demonstrating good internal consistency and test-retest reliability [127].

### Procedure

**Fig 1:**
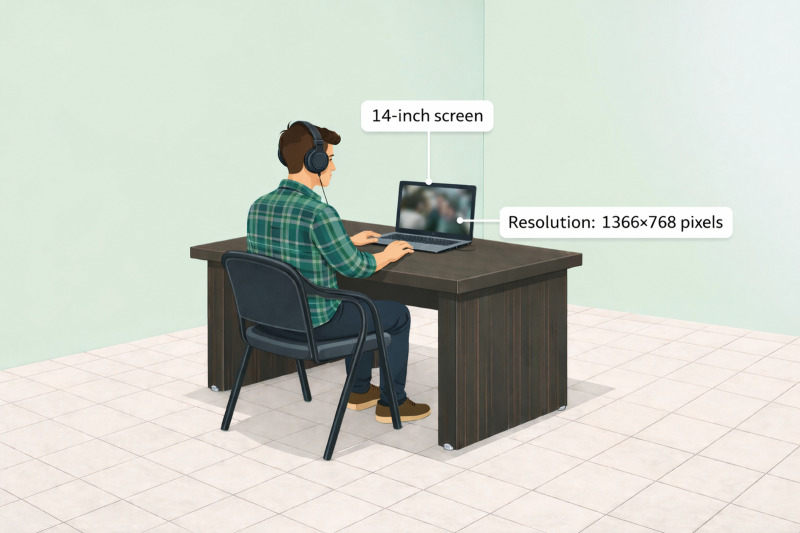
Schematic representation of the experimental setup.

**Fig 2:**
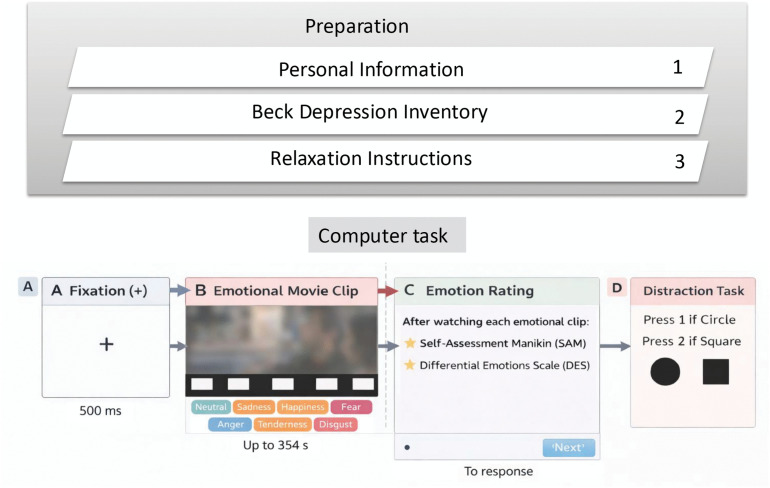
Experimental procedure overview. The procedure consisted of a preparation phase (personal information, Beck Depression Inventory, and relaxation instructions), followed by a computerized task. Each trial included (A) a fixation period (500 ms), (B) an emotional movie clip (up to 354 **s)**, (C) self-report emotion ratings, and (D) a brief distraction task.

The experiment was conducted in a dedicated behavioral laboratory where participants worked independently (see Figs 1 and 2). Each participant was tested in the same controlled environment. Prior to the commencement of the experiment, the room’s lighting was dimmed and participants were given pre-recorded relaxation instructions. These instructions required participants to close their eyes, achieve full-body relaxation, including facial muscles, and engage in deep, regular breathing for a duration of approximately 120 seconds. Upon completion of the relaxation process, the participants promptly commenced the assigned task. Initially, detailed instructions about the test process and how to complete the instrument were displayed on the screen. Participants were encouraged to ask questions if they found any ambiguity in the instructions. A fixation dot was then centrally displayed for 500 ms before each movie was shown. Two neutral movies were consistently shown at the start of the task to familiarize the participants with the task and establish a baseline. The experimental stimuli, in the form of movies, were presented on a 14-inch screen with a resolution of 1366×768 pixels. All clips were presented in full (i.e., they could not be skipped), and therefore each participant watched the complete set of stimuli before proceeding to the post-clip ratings. Each participant was positioned at a 90-degree arc, facing the screen, and provided with individual headphones to ensure an immersive experience. To minimize distractions, participants were instructed to remove any potential devices such as smartphones or smartwatches and to maintain their focus on the screen throughout the experiment. Following each film excerpt, participants were asked to complete computerized questionnaires to assess their emotional state. Based on previous studies’ recommendations [21,23], participants were instructed to (a) express their authentic emotions, rather than what they believed others might expect them to feel in response to the movies, (b) convey the immediate thrill they experienced while viewing the movie, instead of their overall mood throughout the day, (c) disclose if they recognized the movie from the clip, to exclude any clips from our database that had been previously viewed by a minimum of 5% of the participants. In the subsequent stage, participants completed a series of distraction trials. They were instructed to press key 1 or key 2 upon seeing a circle or square, respectively. Participants were then asked to take a deep breath, follow the initial relaxation instructions, and press the “space” button when they were ready to watch the next movie. This procedure, inclusive of the relaxation instructions, was repeated for each film excerpt. Finally, a neutral movie was shown to the participants for emotional recovery, a process that was consistent across all subjects. This was followed by a random display of the movies. The entire experiment was designed and executed using the Python-based Psychopy library on a Windows PC. The experiment was conducted in a controlled setting, free from external interruptions, with only the participant present in the laboratory. This approach ensured a consistent and controlled experimental environment.

It should be noted that the sequence of film presentation was meticulously counterbalanced, adhering to the following criteria: (a) Films sharing identical target emotions were not sequenced back-to-back. (b) Participants were prevented from viewing two films with analogous valence/arousal successively. (c) The order of video clip presentations was randomized. (d) To familiarize participants with the experimental process and establish a baseline, two neutral films were screened at the commencement of the experiment. (e) A neutral film was presented at the conclusion of the experiment to facilitate emotional recovery and the processing of negative stimuli. This approach ensured a balanced and unbiased exposure to the different emotional stimuli throughout the experiment.

### Measures

#### Self-assessment Manikin.

The Self-Assessment Manikin (SAM), a non-verbal pictorial assessment technique that measures pleasure, arousal, and dominance, was introduced by [119]. In this study, we implemented the dimensions of valence and arousal, based on the dimensional structure of emotion as proposed by [129]. These dimensions are frequently used in research contexts [23,68,130,131]. Given its intercultural applicability, SAM is suitable for use across various cultures and countries [132–135]. It serves as an alternative to verbal reporting scales. In our research, we used the computerized version of SAM, developed by [119], which employs a nine-degree scale to evaluate the dimensions of valence and arousal. Participants rated their level of pleasantness/happiness/amusement (coded as 9) or unpleasantness/sadness (coded as 1), as well as arousal (coded as 9) or calmness (coded as 1), after watching each movie during the experiment using a nine-point Likert scale [119]. The responses were based on the participants’ feelings during the movies, rather than what they perceived to be the correct response. The research tool, which uses graphic images to express different emotional states, was easy to use regardless of the participant’s educational level. The Persian version of the experiment was validated by [120] for the Iranian community. Reliability was assessed using a two-week-interval retest method and by calculating Cronbach’s alpha coefficient. The internal consistency of the researcher-developed tool was assessed using Cronbach‘s alpha, yielding values of 0.89 and 0.83 for the pleasantness and arousal dimensions, respectively. These results indicate an acceptable level of reliability.

#### Differential emotions scale.

In our endeavor to evaluate discrete emotional dimensions following the viewing of each video clip, we have, for the first time to our knowledge, developed and validated a Differential Emotions Scale in Persian within Iranian society, drawing upon the insights from previous studies [21,30,46,115,116]. Our choice of the DES was motivated by its widespread use as a self-report scale for capturing discrete emotional feelings [30,83,136,137]. Specifically, we adapted and validated this Persian version of the DES, which had previously been successfully employed in validating emotional film stimuli in various languages, including English [116,138] and French [30,83]. The DES comprises groups of emotional adjectives, including descriptors such as ‘interested, concentrated, alert,’ ‘joyful, happy, amused,’ ‘sad, downhearted, blue,’ ‘angry, irritated, mad,’ ‘fearful, scared, afraid,’ ‘anxious, tense, nervous,’ ‘disgusted, turned off, repulsed,’ ‘disdainful, scornful, contemptuous’, ‘surprised, amazed, astonished,’ ‘warm-hearted, gleeful, elated,’ ‘loving, affectionate, friendly,’ ‘guilty, remorseful,’ ‘moved,’ ‘ satisfied, pleased,’ ‘calm, serene, relaxed,’ and ‘ashamed, embarrassed.’ Participants were asked to rate the intensity of their emotions for each item on a 7-point scale (ranging from ‘not at all‘ to ‘very intense’) after viewing each film clip. To maintain the flow of this paper, we have detailed the methods and analyses used for the validation and reliability of this Persian version of DES in S1 Appendix. Additionally, the Persian version of the DES is freely accessible to researchers via the open science framework at https://osf.io/2td5n/.

### Data analysis

The data analysis involved several statistical procedures to evaluate the effectiveness of the video clips in eliciting specific emotional responses across multiple dimensions. To assess differences in emotional responses elicited by different categories of video clips, within-subjects repeated measures analyses of variance (ANOVA) were performed. Following the ANOVA, post-hoc multiple pairwise comparisons were carried out using Bonferroni’s correction to identify specific differences between the emotional categories. The results of these post-hoc tests provided insights into which categories significantly differed from one another in terms of the emotional dimensions under study. Significance levels were set at various thresholds to capture a range of statistical strengths. All analyzes were performed using R, with effect sizes calculated to provide additional insights. Exploratory analyses also examined potential gender differences. This approach validated our database’s effectiveness in an Iranian cultural context.

### Transparency and openness

We report how we determined our sample size, all data exclusions, all manipulations, and all measures in the study, in accordance with the Journal Article Reporting Standards (JARS; Appelbaum et al., 2018). All data, analysis code, and materials for this study are publicly available on the Open Science Framework (OSF) and can be accessed at https://osf.io/2td5n/. Data were analyzed using R, version 4.0.0 (R Core Team, 2020), and the package ggplot, version 3.2.1 (Wickham, 2016). This study‘s design and hypotheses were not preregistered.

### Inclusivity in global research

Additional information regarding the ethical, cultural, and scientific considerations specific to inclusivity in global research is included in the Supporting information (S1 Checklist).

## Results

The findings are focused on these key questions: Which categories of video clips elicited the most potent levels of emotional arousal? Which categories of video clips are most successful in inducing significant levels of emotional valence? Are the video clips capable of inducing distinct positive and negative affective states? Are we able to elicit differentiated emotional feeling states using the video clips? Are there any gender-based differences in emotional responses? What criteria should be used to select the most effective video clips from the database? In the following, we aim to thoroughly investigate each of these questions within the Iranian community and subsequently provide a separate report on the findings obtained for each one.

### Which categories of video clips elicited the most potent levels of emotional arousal?

**Fig 3:**
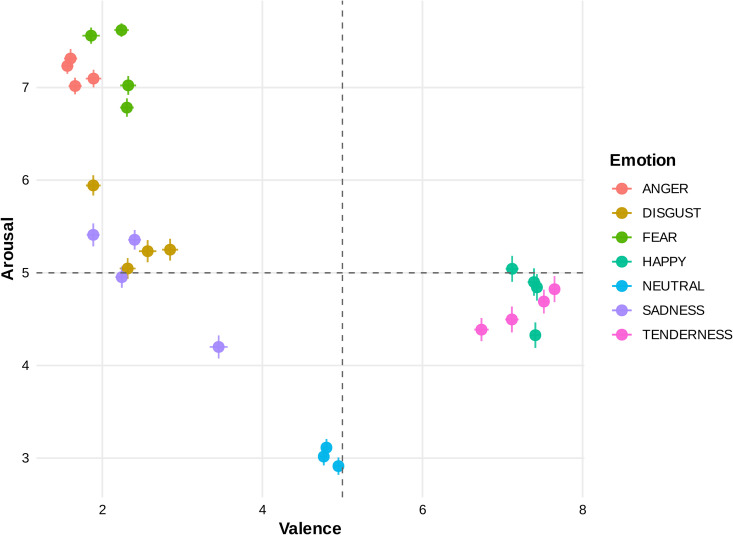
Subject-level analysis of continuous emotional dimensions. This figure illustrates the analysis of emotional dimensions at the subject level. The horizontal axis denotes the valence dimension, reflecting the spectrum of positive to negative emotions. The vertical axis measures the arousal dimension, indicating the level of emotional activation or intensity. The mean and standard error are shown for each dimension.

**Fig 4:**
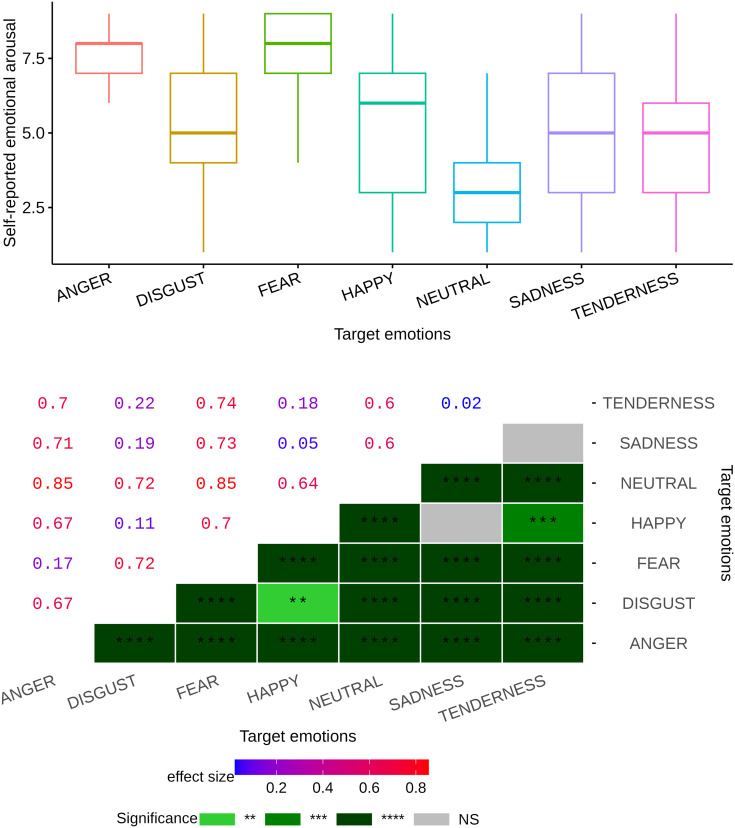
Self-reported emotional arousal levels across various emotional categories at the individual level, with a heatmap displaying Bonferroni pairwise comparison p-values and their corresponding effect sizes. Significance codes: NS (non-significant), ‘****‘ (<0.0001), ‘***‘ (<0.001), ‘**‘ (<0.01), ‘*‘ (<0.05).

We first examined the self-reported arousal scale corresponding to each video clip across a range of emotional categories (see Fig 3). The statistical results of the impact of these categories on this particular arousal scale are shown in Fig 4, which encapsulates the metrics such as Bonferroni’s correction p-values and effect sizes visually in a heatmap. The data revealed a strong main emotional effect ($p<10^{-6}$, $\eta^{2}=0.43$). Upon closer examination of Fig 4, we can observe that the Bonferroni post hoc test highlighted a profound level of significance in all pairwise comparisons associated with the neutral category, evidenced by a p-value less than $10^{-6}$ (additional details and corresponding effect sizes can be found in Fig 4). Furthermore, a significant difference was observed in all pairwise comparisons among emotional categories, except for the pairs HAPPINESS–SADNESS and SADNESS–TENDERNESS (see Fig 4 for more information). These findings suggest that all emotional movies elicited higher levels of self-reported arousal compared to neutral movies, with fear-inducing movies generating the most intense arousal levels, while clips from movies in the sadness category produced lower arousal levels than other negative emotional films, though still higher than neutral films. This underscores the differential impact of various emotional categories on arousal levels.

### Which categories of video clips are most successful in inducing significant levels of emotional valence?

**Fig 5:**
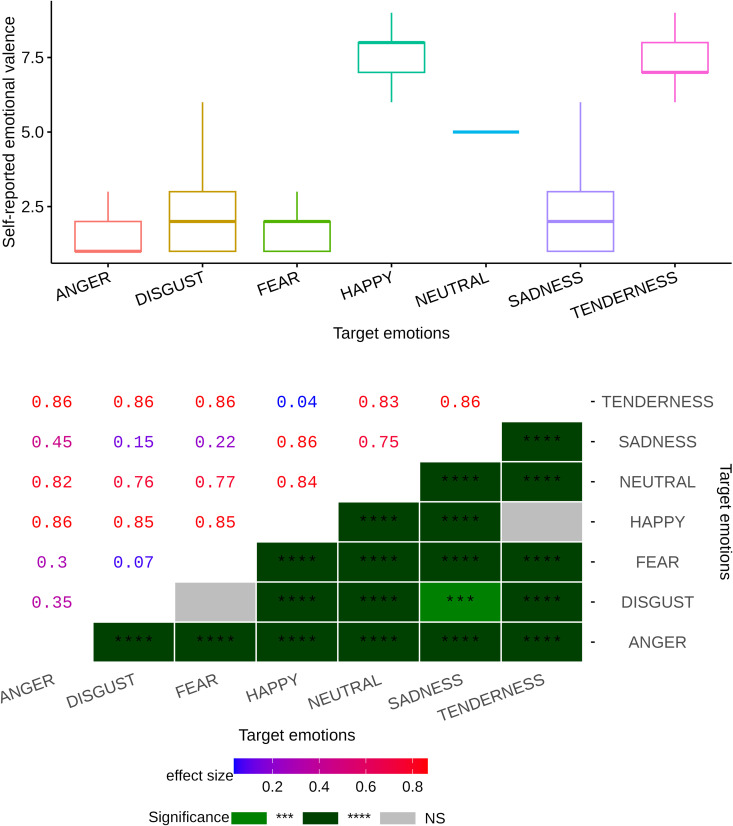
Subject-level self-reported emotional valence across various emotional categories, with a heatmap displaying Bonferroni pairwise comparison p-values and their corresponding effect sizes. Significance codes: NS (non-significant), ‘****‘ (<0.0001), ‘***‘ (<0.001), ‘**‘ (<0.01), ‘*‘ (<0.05).

Here, as our second question, we explored how different emotional video clips impact valence, another emotional dimension recognized through continuum theory, where a high valence rating signifies the pleasantness and a low valence rating indicates the unpleasantness of that particular category of movies. The measurement results for each target emotion category are graphically represented in Figs 3 and 5, which statistically demonstrates a strong main emotional impact ($p<10^{-6}$, $\eta^{2}=0.71$). As shown in the heatmap in Fig 5, in addition to all Bonferroni pairwise comparisons associated with the neutral genre being high significant at $p<10^{-6}$, the pairwise comparisons of all emotional genres (except for FEAR–DISGUST, and HAPPINESS–TENDERNESS pairs) are statistically significant (see Fig 5 for details and corresponding effect sizes). The findings suggest that both positive emotional film categories (i.e., HAPPINESS, which produces higher levels of valence, and TENDERNESS) were successful in creating stronger levels of self-reported valence compared to neutral and negative films. Furthermore, the study reveals that the ANGER category of video clips resulted in the lowest level of valence. Therefore, it can be concluded that emotional films in the positive and negative genres have been successful in extracting positive and negative emotions, respectively.

### Are the video clips capable of inducing distinct positive and negative affective states?

To test the discriminant induction validity of the positive-negative affective states across our categories of emotional video clips, we implemented a procedure to establish two robust/reliable measures. These measures, termed as positive and negative composite scores, represent Positive Affect (PA) and Negative Affect (NA) for each emotional condition. They were derived by averaging the classes of DES items. The positive composite score incorporated six items: “joyful, happy, amused”; “warm-hearted, gleeful, elated”; “loving, affectionate, friendly”; “interested, concentrated, alert”; “satisfied, pleased” “calm, serene, relaxed”. On the other hand, the negative composite score encompassed ten items: “fearful, scared, afraid”; “anxious, tense, nervous”; “moved”; “angry, irritated, mad”; “Ashamed, embarrassed” “sad, downhearted, blue”; “surprised, amazed, astonished”; “guilty, remorseful”; “disgusted, turned off, repulsed”; “disdainful, scornful, contemptuous”. Our results have strongly validated these two composite measures in the Iranian culture, mirroring findings from previous studies in other cultures (e.g., [23]). In particular, all Cronbach‘s alphas in our study exceeded 0.60, signifying a strong internal consistency for our scales, which aligns with Schmitt’s criterion [139] that deems a composite measure satisfactory with a value of 0.50, thereby highlighting the cross-cultural applicability of these composite measures. In response to these findings, our data analysis here is designed to address the following question: To what degree were the films categorized under positive and negative emotional states successful in eliciting the corresponding emotions? More precisely, we seek to determine whether the films, previously classified under positive and negative emotional categories by preceding studies, receive analogous ratings from Iranian participants. To this end, we conducted two separate statistical tests to examine the effect of the video clips category on PA and NA scores across all 27 video clips viewed by the participants.

**Fig 6:**
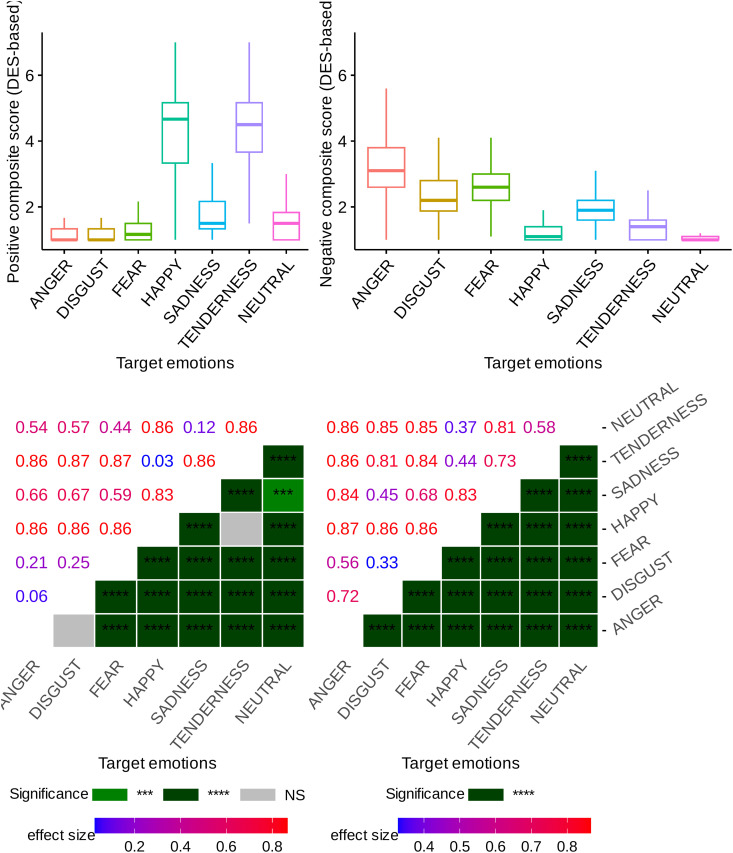
Subject-level self-reported positive (displayed on the left) and negative (shown on the right side) composite scores (DES-based) across various emotional categories, with a heatmap displaying Bonferroni pairwise comparison p-values and their corresponding effect sizes. Significance codes: NS (non-significant), ‘****‘ (<0.0001), ‘***‘ (<0.001), ‘**‘ (<0.01), ‘*‘ (<0.05).

The analysis of the data, with a focus on positive and negative composite scores, reveals a significant main effect of video clip categorization on PA ($p<10^{-6}$, $\eta^{2}=0.68$) and NA ($p<10^{-6}$, $\eta^{2}=0.74$), independently. We executed the corresponding post-hoc tests using Bonferroni pairwise comparisons to evaluate if the ratings for negative and positive affects differed between positive and negative video clips. Regarding the scores for positive affect, all positive video clips received significantly higher ratings compared to the negative video clips, evidenced by a p-value less than p<0.0001 (additional details and corresponding effect sizes can be found in Fig 6). Furthermore, the Bonferroni pairwise comparisons between all pairs of emotional categories, excluding ANGER–DISGUST and HAPPINESS–TENDERNESS, were statistically significant according to the post-hoc tests (see Fig 6 for more information). As depicted on the left side of Fig 6, the video clips from the HAPPINESS and TENDERNESS categories achieved the highest scores in terms of positive emotions. On the other hand, all negative video clips scored statistically higher than the positive video clips in terms of negative affect scores (p<0.0001, see Fig 6). As illustrated on the right side of Fig 6, video clips in the ANGER, FEAR, and DISGUST categories received the highest scores for the negative affect items. Additionally, the Bonferroni post-hoc pairwise comparison revealed a significant difference between each pair of emotions and all pairs within the negative genre (see Fig 6 for detailed information and corresponding effect sizes).

### Are we able to elicit differentiated emotional feeling states using the video clips?

**Fig 7:**
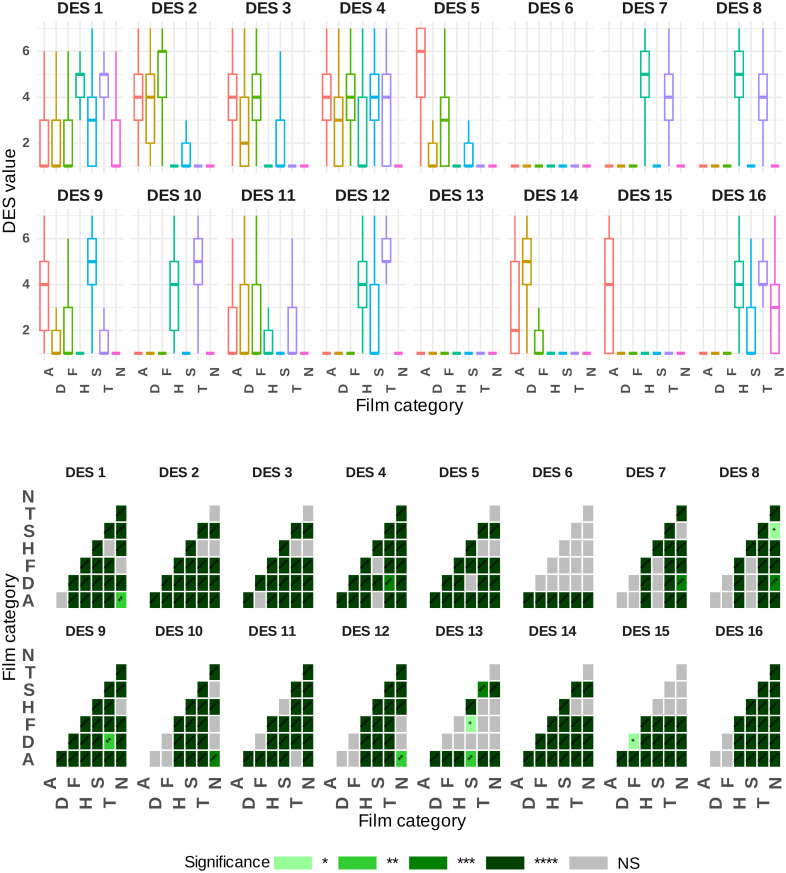
Subject-level self-reported emotional profiles based on DES elements across various discrete emotional categories of video clips, with a heatmap displaying Bonferroni pairwise comparison p-values. Significance codes: NS (non-significant), ‘****’ (<0.0001), ‘***’ (<0.001), ‘**’ (<0.01), ‘*’ (<0.05). Emotion abbreviations: A = anger, D = disgust, F = fear, H = happy, S = sadness, T = tenderness, N = neutral.

**Fig 8:**
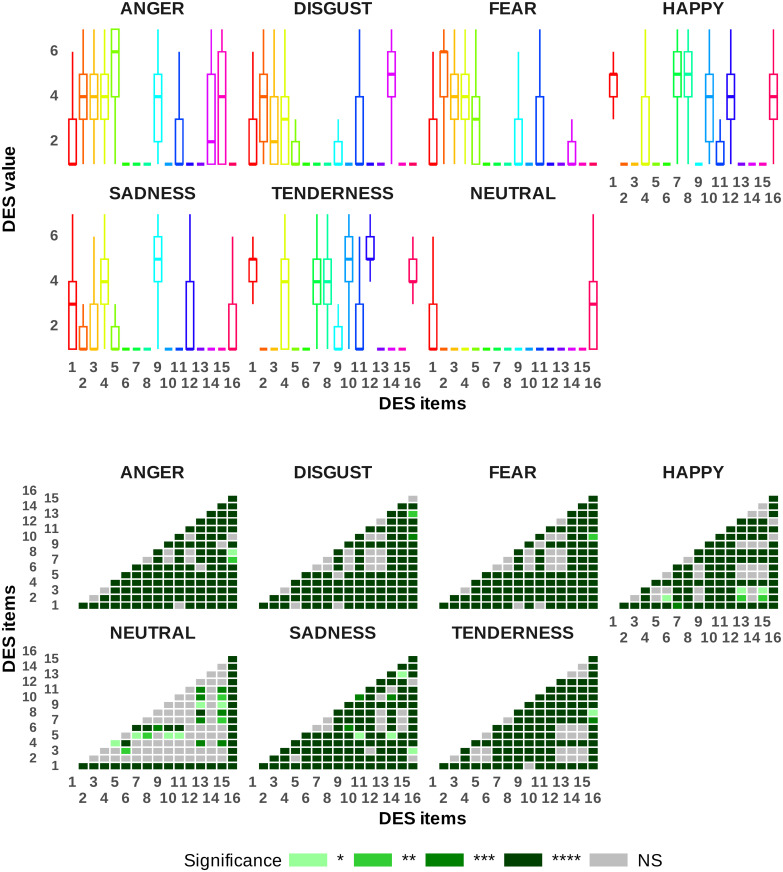
Subject-level self-reported emotional profiles based on DES elements across various discrete emotional categories of video clips, with a heatmap displaying Bonferroni pairwise comparison p-values. Significance codes: NS (non-significant), ‘****’ (<0.0001), ‘***’ (<0.001), ‘**’ (<0.01), ‘*’ (<0.05).

Perhaps it can be said that the most important feature that should be thoroughly examined in the context of emotional validation studies similar to this study is the elicitation of distinctly different emotional feeling states using the desired video clips. Due to the large sample size, this aspect can be investigated here based on the deep analysis of the DES. Specifically, it can explore the fundamental hypothesis of whether self-reported emotional profiles are modulated by video clip categorization. From a statistical perspective, an analysis of variance on repeated measures of 7 × 16 results revealed a strong and significant interaction between video clip categorization and DES elements, clearly supporting the proposed hypothesis ($p<10^{-16}$, $\eta^{2}=0.46$). Detailed statistical results on discrete emotional regulation by each induced video clip category are presented in Figs 7 and 8, including corrected p-values (using the Bonferroni method) and effect sizes visually in heatmaps. A closer examination of the heatmaps in Figs 7 and 8 reveals that most Bonferroni pairwise comparisons between the DES items were highly significant at p<0.001 (see Figs 7 and 8 for more details and corresponding effect sizes). These findings confirm the anticipated differentiation between emotional states. To provide further insight, the results can be analyzed by first targeting the specific DES item for each discrete emotional state. For instance, ANGER corresponded to DES item 5: “*angry, irritated, mad*,” FEAR to item 2: “*fearful, scared, afraid*,” TENDERNESS to item 12: “*loving, affectionate, friendly*,” SADNESS to item 9: “*sad, downhearted, blue*,” HAPPINESS to item 8: “*joyful, amused, happy*,” DISGUST to item 14: “*disgusted, turned off, repulsed*,” and NEUTRAL to item 16: “*calm, serene, relaxed*.” Subsequently, we conducted a set of six Bonferroni pairwise comparisons for each emotional category of video clips to examine the differences between the target state (associated with the chosen DES item) and each non-target state, as detailed in Figs 7 and 8. For example, in the category of SADNESS video clips, we zoom in on the statistical results of the Bonferroni pairwise comparisons of the specific DES item (‘‘*sad, downhearted, blue*”) with the six non-target items, which are targets for the other six emotional categories (e.g., ANGER, TENDERNESS, etc.). The statistical analyses in Figs 7 and 8 clearly show that all comparisons are highly significant, thereby supporting the hypothesis and the expected differences between the target states.

### Are there any gender-based differences in emotional responses?

**Fig 9:**
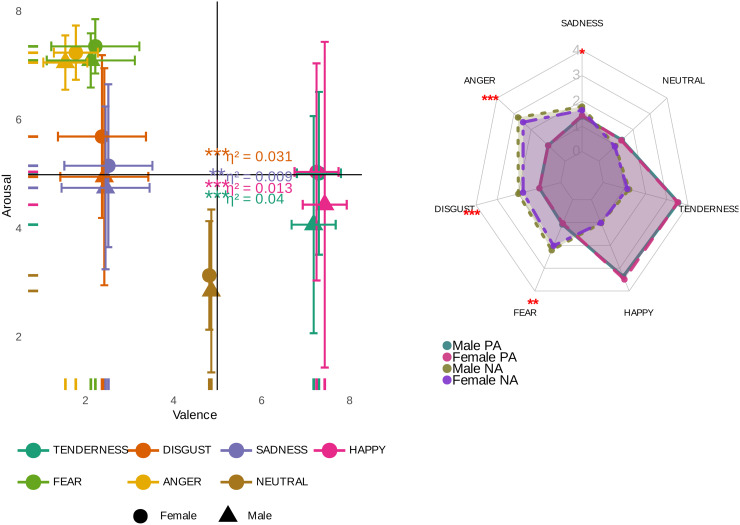
Gender-based differences in continuous emotional dimensions, which include arousal, valence, and positive and negative affects across various emotional categories of video clips, with Bonferroni pairwise comparison p-values and their corresponding effect sizes. Left panel: The effect of gender on arousal and valence. Right panel: Gender differences in positive & negative emotions. Significance codes: NS (non-significant), ‘****‘ (<0.0001), ‘***‘ (<0.001), ‘**‘ (<0.01), ‘*‘ (<0.05).

Here, we have given particular attention to the role of gender in influencing continuous emotional dimensions, which include arousal, valence, positive and negative affects, and discrete emotions based on the DES elements. These were initially evaluated in Subsections 1 and 2 without gender as an additional factor. However, upon incorporating gender into our statistical analyses, we found that while it does not significantly influence valence (p>0.25, $\eta^{2}=0.0001$), it may have a significant main effect on the emotional arousal scale within the Iranian population ($p<10^{-4}$, $\eta^{2}=0.005$), see Fig 9 for more details. To further investigate this effect, we conducted post-hoc tests using Bonferroni pairwise comparisons. Our observations revealed a significant difference between women and men in their emotional arousal responses during the presentation of video clips designed to induce feelings of HAPPINESS, TENDERNESS, SADNESS, and DISGUST (see Fig 9 for more details and corresponding effect sizes). Specifically, women probably exhibited significantly higher levels of emotional arousal compared to men. However, it is crucial to highlight that despite these significant differences, the corresponding effect sizes were relatively small, suggesting a negligible impact (see Fig 9).

On the other hand, the investigation of the effect of gender on positive and negative emotions based on the composite DES scores showed no significant main effect on positive emotions (p>0.5, $\eta^{2}=0.0001$), but identified a significant main effect on negative emotions ($p<10^{-4}$, $\eta^{2}=0.001$), see Fig 9 for more details. More specifically, based on Bonferroni pairwise comparisons, it was observed that gender creates a significant difference on the negative emotion scale during the viewing of a set of video clips inducing feelings of SADNESS, FEAR, ANGER, and DISGUST (see Fig 9 for more details). However, we believe that given the extremely low effect sizes (<0.01), this effect can be completely negligible.

**Fig 10:**
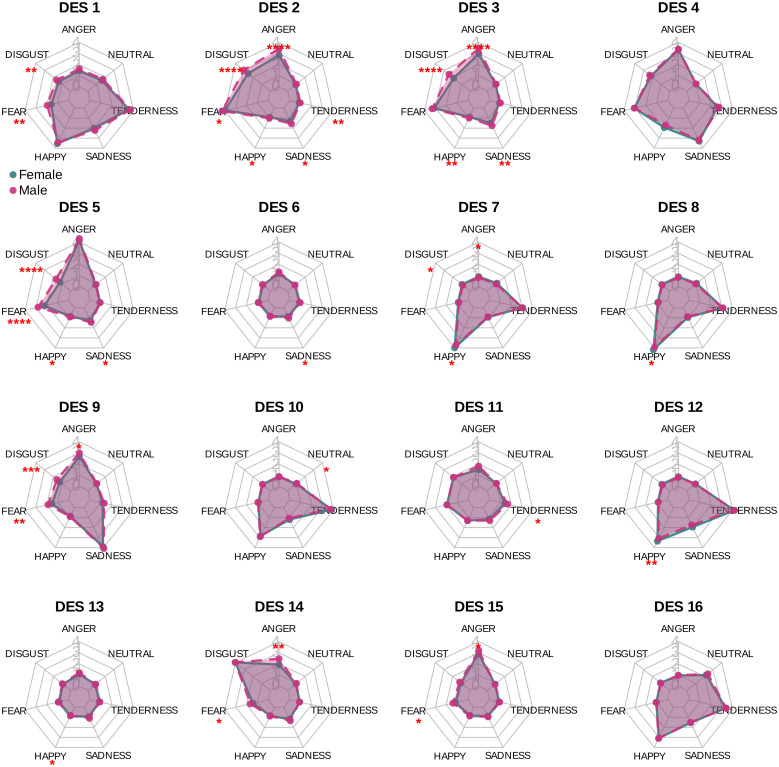
Gender-based differences in discrete emotions based on the DES elements across various emotional categories of video clips, with Bonferroni pairwise comparison p-values. Significance codes: NS (non-significant), ‘****‘ (<0.0001), ‘***‘ (<0.001), ‘**‘ (<0.01), ‘*‘ (<0.05).

In the final gender analysis, a significant main effect was found when examining the impact of gender on the DES items ($p<10^{-16}$, $\eta^{2}=0.003$). Based on Bonferroni pairwise comparisons, it was observed that gender significantly impacts various DES items under different emotional conditions induced by video clips (see Fig 10 for details). Specifically, gender significantly influenced:

- DES 1 during the viewing of clips eliciting feelings of DISGUST (p<0.01, $\eta^{2}=0.43$) and FEAR (p<0.002, $\eta^{2}=0.53$),
- DES 2 during clips inducing feelings of ANGER ($p<10^{-6}$, $\eta^{2}=0.55$), DISGUST ($p<10^{-5}$, $\eta^{2}=0.36$), FEAR (p<0.02, $\eta^{2}=0.44$), HAPPINESS (p<0.01, $\eta^{2}=0.1$), SADNESS (p<0.01, $\eta^{2}=0.28$), and TENDERNESS (p<0.007, $\eta^{2}=0.29$),
- DES 3 during the viewing of video clips inducing feelings of ANGER ($p<10^{-5}$, $\eta^{2}=0.45$), DISGUST ($p<10^{-6}$, $\eta^{2}=0.4$), HAPPINESS (p<0.002, $\eta^{2}=0.17$), and SADNESS (p<0.004, $\eta^{2}=0.27$),
- DES 5 during the viewing of video clips inducing feelings of DISGUST ($p<10^{-5}$, $\eta^{2}=0.41$), FEAR ($p<10^{-5}$, $\eta^{2}=0.41$), HAPPINESS (p<0.02, $\eta^{2}=0.18$), and SADNESS (p<0.03, $\eta^{2}=0.2$),
- DES 6 during clips inducing feelings of SADNESS (p<0.03, $\eta^{2}=0.47$),
- DES 7 during clips eliciting feelings of ANGER (p<0.02, $\eta^{2}=0.39$), DISGUST (p<0.03, $\eta^{2}=0.44$), and HAPPINESS (p<0.02, $\eta^{2}=0.39$),
- DES 8 during clips inducing feelings of HAPPINESS (p<0.02, $\eta^{2}=0.35$),
- DES 9 during the viewing of video clips inducing feelings of ANGER (p<0.02, $\eta^{2}=0.26$), DISGUST (p<0.001, $\eta^{2}=0.3$), and FEAR (p<0.005, $\eta^{2}=0.32$),
- DES 10 during the viewing of NEUTRAL clips (p<0.03, $\eta^{2}=0.2$),
- DES 11 during clips eliciting feelings of TENDERNESS (p<0.04, $\eta^{2}=0.56$),
- DES 12 and DES 13 during clips inducing feelings of HAPPINESS (DES 12: p<0.005, $\eta^{2}=0.26$; DES 13: p<0.02, $\eta^{2}=0.31$),
- DES 14 and DES 15 during the viewing of video clips inducing feelings of ANGER (DES 14: p<0.003, $\eta^{2}=0.56$; DES 15: p<0.04, $\eta^{2}=0.21$) and FEAR (DES 14: p<0.04, $\eta^{2}=0.57$; DES 15: p<0.02, $\eta^{2}=0.44$).

Based on the results presented here, we observe that around 30% of all possible cases demonstrated statistical significance (see Fig 10), indicating the impact of gender differences on DES items. However, it is essential to note that our investigation into the question in Subsection 4, was primarily focused on six out of the 16 possible DES items, which were considered as the main indicators of discrete emotions. Upon examining these six items, it was found that none of them reached statistical significance at a reasonable level of significance, such as p<0.01. Consequently, the gender differences observed in this study do not contribute any bias or effects to the analyses once the gender factor is excluded.

### What criteria should be used to select the most effective video clips from the database?

In mood induction studies, the selection of the most effective mood-inducing tools is of paramount importance. Researchers often formulate their questions and hypotheses around specific emotional variables, recognizing that not all variables are equally relevant. To address this, within the extensive collection of mood-induction video clips thoroughly examined here, we classified and rated them based on various distinct discrete and dimensional emotional variables. Beyond ranking these mood-inducing clips according to the self-reported experiences of individuals at a particular emotional state, we have also developed a 10-point rating system for these clips, taking into account all factors such as valence, arousal, each of the seven discrete emotions, and the balance of negative/positive affects. This methodological approach provides researchers with the capability to efficiently identify the most impactful video clips on a specific emotional dimension within our database.

The emotional rating system developed was grounded on a comprehensive set of 25 fundamental criteria, utilizing a video clip-level analysis lens. The procedure was as follows: First, a measurement matrix for all video clips in our database was computed based on the mean and standard deviation of individuals’ emotional variable scores related to arousal, valence, PA, and NA for each video clip. It is important to note that for the calculation of the mean and standard deviation of PA and NA scores across individuals for each video clip, each video clip was first evaluated on all 16 DES items. Then, based on the method described in Subsection 3, two video clip-level coefficients for the positive and negative composite scores were derived from the averaged DES item scores. In the second step, we tried to complete this four-criteria system by adding a six-criteria class named with discreteness coefficients each corresponding to the emotional categories (ANGER, FEAR, HAPPINESS, DISGUST, SADNESS, and TENDERNESS), with the explicit aim of classifying video clips based on the distinct levels of emotional states they elicit. To calculate each of the six discreteness coefficient criteria, the mean DES score of the scale targeting one specific emotion was subtracted from the averaged mean scores of the scales targeting the other five emotions. Finally, we completed the ten-criterion system mentioned thus far by adding a fifteen-criterion class named with mixed feelings (MF) coefficients, aiming to classify video clips based on a quantitative estimate of a more complex structure of human emotions, i.e., the mixed/composite emotions elicited pairwise by each video clip. Mathematically, there are various approaches to quantifying an estimation criterion for mixed feelings, ranging from simple additive, multiplicative, and minimum methods to more complex approaches such as dimensional scaling and machine learning algorithms like Principal Component Analysis (PCA), Independent Component Analysis (ICA), Multidimensional Scaling (MDS), etc. For the sake of simplicity and to maintain focus on the primary objective of this paper, we employed a multiplicative approach where the degree of the MF score is calculated based on the product of two elicited emotional scores (e.g., FEAR and DISGUST) associated with the same video stimulus. This formula is such that higher scores indicate stronger mixed feelings. If either emotion is low, the product—and consequently the MF coefficient—is low, and if both emotions are high, the product is higher, indicating stronger mixed emotions.

**Fig 11:**
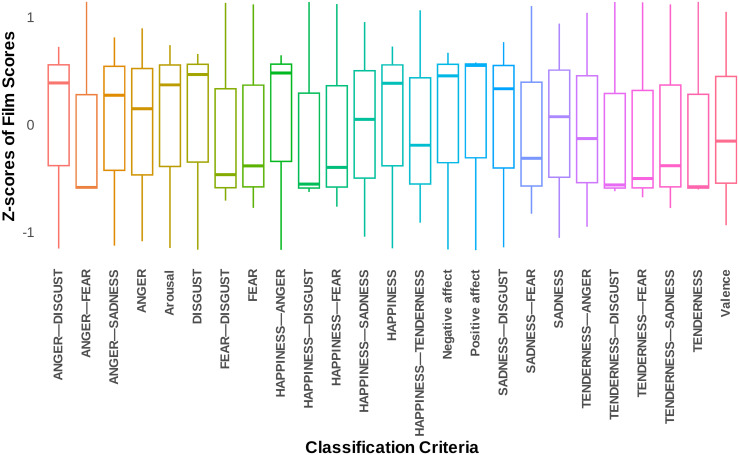
Shortlist distribution across criteria. This figure illustrates the distribution of shortlisted scores according to various criteria. The horizontal axis denotes the different classification criteria, while the vertical axis indicates the standardized scores, or Z-scores, applicable to all criteria.

**Fig 12:**
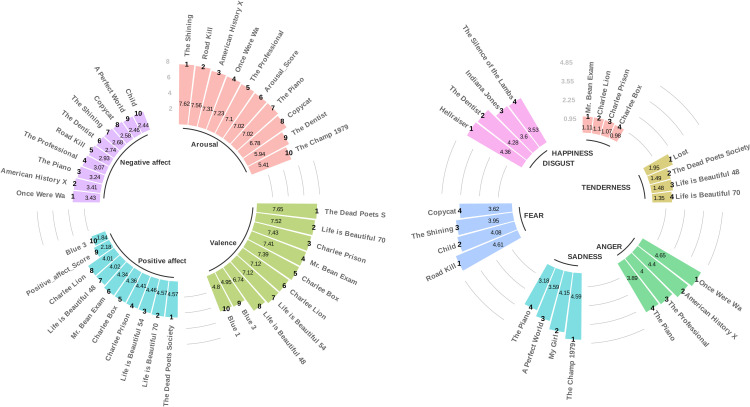
Top-ranked video clips by criterion. This figure displays the video clips that rank highest, known as the shortlists, for each specific criterion each labeled with the emotional criterion’s score and its rank within the category. On the left, clips are aligned with continuous dimensional emotional variables, while on the right, they correspond to discrete, distinct emotional variables. These clips are selected to elicit the strongest response in the targeted emotional variable.

**Fig 13:**
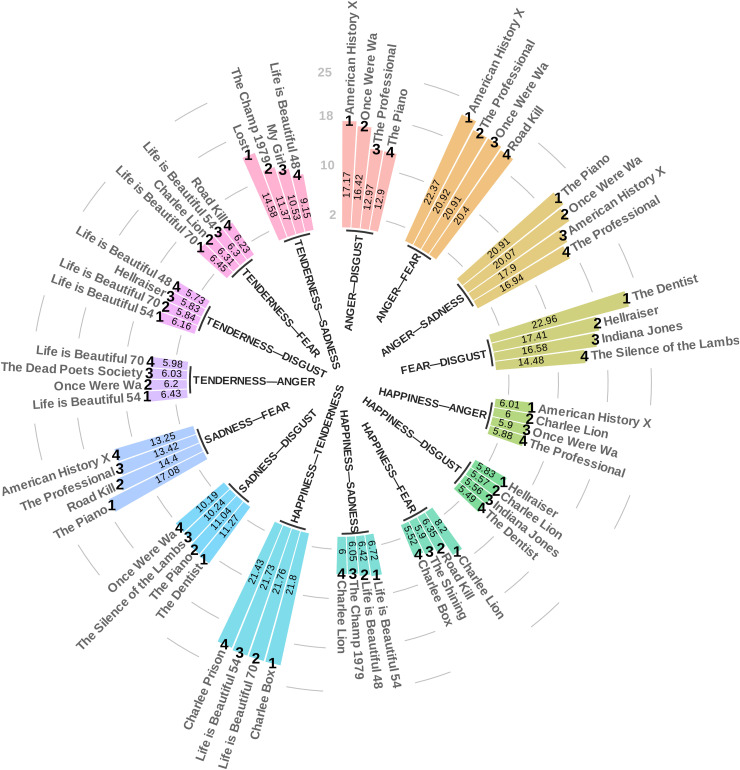
Top-ranked video clips by mixed feelings criteria. This figure displays the video clips that rank highest, known as the shortlists, for each specific MF coefficient, each labeled with the MF’s score and its rank within the category. These clips are selected to elicit the strongest response in the mixed/composite emotional variable.

Thus, our emotional rating system is complete with the 25 fundamental criteria discussed above, providing a comprehensive framework for the classification and analysis of emotional responses elicited by video clips. The measurement matrix, which corresponds to all video clips in our database with the 25 fundamental criteria, is available as a table on the Open Science Framework (https://osf.io/2td5n/). As the final step in our video clip-level analysis, we identified the highest-ranking video clips based on 25 final criteria, separately for each criterion, to indeed have the video clips that induce the highest level of the targeted emotional variable. To ensure the robustness of the shortlists, we conducted several additional statistical analyses, including testing the hypothesis that each video clip in the shortlist for a specific criterion significantly differs from the average of the entire video clip database. The results were all significant with a p-value less than $10^{-6}$, indicating a very high level of efficiency for each criterion for each video clip in the shortlist. Additionally, within each shortlist, we performed an additional outlier analysis based on the conventional criterion of 1.5 times the interquartile range (IQR) from the first (Q1) and third quartiles (Q3) to identify any specific video clips that were significantly less or more effective than others in the shortlist, but no such cases were found, and all results were consistent (see Fig 11). Finally, the shortlists obtained for each of the 25 final criteria can be seen in detail in Figs 12 and 13.

## Discussion

The current study aimed to develop and validate the effectiveness of a comprehensive and newly developed emotional video clip database for emotion induction across Iranian culture and society according to different validity criteria. This initiative fills a significant gap in emotion research, as previous efforts have predominantly focused on Western populations, often overlooking the intricate cultural factors that shape emotional experiences. By evaluating the effectiveness of 27 carefully selected video clips, this study provides a robust tool for emotion researchers within and beyond Iran, contributing to the growing body of literature on culturally sensitive emotion induction techniques [22–24,60,84–87]. To that end, several questions were analyzed and the validation process involved a multifaceted analysis based on several validity criteria, e.g., general arousal, valence, seven criteria for emotional discreteness (ANGER, FEAR, HAPPINESS, DISGUST, SADNESS, TENDERNESS, and NEUTRAL), two dimensions of positive and negative effects based on DES scores, mixed-feeling scores (as measured by the DES), and gender differences across these dimensions.

The validation of emotional stimuli in this study was meticulously designed to address both the breadth and depth of emotional experiences. In particular, our approach to validating emotional stimuli was grounded in a robust theoretical framework, incorporating both basic emotion theories and dimensional models. By drawing on two major theoretical approaches to emotion—the basic emotion approach [89,115,140] and the dimensional approach [16,130]—we ensured that our assessment captured the multifaceted nature of emotional responses. The basic emotion approach, which categorizes emotions into distinct types such as happiness, sadness, anger, and fear, has been a widely accepted framework in emotion research [89,115]. In contrast, the dimensional approach, which emphasizes the continuous nature of emotional experiences along axes such as arousal and valence, offers a more nuanced understanding of how emotions are experienced and reported [16,130]. This dual approach ensured a comprehensive evaluation of the emotional efficacy of the video clips. Given the importance of culture and language in emotion induction, the study’s focus on an Iranian sample addresses a gap in existing research, where such databases are often developed with little consideration for cultural specificity. Previous studies, such as those by [22–24,60,84–87], have emphasized the role of culturally relevant stimuli in emotion induction, yet few have systematically developed a database within a specific cultural context. Our findings are consistent with previous studies that have demonstrated the utility of combining these approaches to provide a comprehensive assessment of emotional states [23,24,60,63,84–87].

One of the key contributions of this study is its focus on the cultural specificity of emotion induction techniques. Culture and language play crucial roles in shaping how individuals experience and express emotions, as evidenced by cross-cultural research in emotion psychology [141,142]. These factors are often underrepresented in emotion research, which tends to prioritize universality over cultural variability [142,143]. In the Iranian context, where cultural and linguistic diversity is profound, it was essential to create a database that could reflect and accommodate this diversity. In this context, the use of video clips, unlike static images, words, or music, offers a rich, dynamic medium where emotions can be elicited through the intricate interplay of audiovisual elements, narrative content, and social cues. This makes them particularly effective for capturing the complexity of emotional experiences within a specific cultural framework, as demonstrated by our findings. Previous research, including studies like those by [22–24,60,84–87], has shown that emotional stimuli can have different effects depending on cultural context. Our findings align with these studies, suggesting that while some emotions, such as fear, may be universally experienced, others, such as tenderness, may be more culturally specific [23,142].

The ethnic, cultural, and linguistic diversity of Iran presented both a challenge and a unique opportunity for this research. With a sample of 300 individuals representing Persian, Kurdish, and Turkish speakers, we were able to assess how these diverse groups respond to emotional stimuli. It is important to note that while these participants come from different linguistic backgrounds, they are all fluent in Persian, which is the official language of Iran and is widely used in schools, official media, and other formal settings. This approach not only enhances the ecological validity of our findings but also provides insights into the potential universality or specificity of emotional responses across different cultural groups within Iran. Similar findings have been reported in studies examining emotion induction in culturally diverse populations [143,144]. The inclusion of non-Persian clips in our study in an Iranian context allowed us to explore cross-cultural similarities and differences in how foreign stimuli are perceived in the Iranian context. Our findings align with previous cross-cultural studies [23,24,60,84–87,145], indicating that the selected clips effectively induced the intended emotional states among Persian participants.

The results demonstrated that the clips used within the subgroups were more effective in eliciting the intended emotional states during testing, as detailed in the previous section. The video clips successfully triggered distinct emotional responses, showing significant distinctions between positive and negative affects, while also maintaining a high degree of discreteness in evoking the seven target emotional states within each category of video clip. Our results indicated that all emotional video clips, particularly those designed to elicit fear, were highly effective in generating higher levels of self-reported arousal compared to neutral films. Fear-inducing video clips, in particular, produced the most intense arousal levels ($p<10^{-6}$), underscoring the potency of fear as an emotion that can transcend cultural boundaries. This is consistent with the findings of [22–24,86], who noted that fear is one of the most reliably elicited emotions across different populations. Positive emotions such as happiness and tenderness also resulted in significantly higher valence scores compared to neutral and negative emotions, with anger-inducing films yielding the lowest valence levels. The majority of comparisons, grounded in these criteria, were significant and validated the anticipated differentiation between target states. However, the distinction between fear and anger was less pronounced during the anger induction phase, suggesting a potential overlap in the emotional experiences of these two states. This finding highlights the complexity of emotional experiences and the potential for emotions to coexist or interact in ways that are not fully captured by traditional models [129,146]. Further analysis of the anger-inducing films revealed that only ’Once Were Warriors’ significantly differentiated between fear and anger, suggesting that some films may evoke mixed emotional responses, a phenomenon also reported in other studies [23,62]. This finding highlights the need for further research into the nuances of emotion induction, particularly in culturally diverse settings.

Gender differences in emotional responses were another focal point of this study. Consistent with a substantial body of research, including studies by [99,147], our findings indicated that women reported higher levels of emotional arousal than men, particularly in response to negative emotions. However, it is important to emphasize that these gender-related effects were very small in magnitude ($\eta^{2}<\mathrm{.01}$) and should therefore be interpreted cautiously as exploratory differences with limited practical significance, likely facilitated by the large sample size. This gender difference in emotional arousal is a well-documented phenomenon and has been attributed to a combination of neurobiological and sociocultural factors [99,147–149]. Neurobiologically, it has been suggested that hormonal differences, particularly in the functioning of the amygdala, may underlie these gender differences in emotional reactivity [147,150,151]. Socioculturally, gender roles and expectations may influence how men and women experience and express emotions [99,148,149]. Moreover, our study revealed significant gender differences in the experience of specific negative emotions, particularly fear, sadness, anger, and disgust. Women were found to report higher levels of these discrete emotions compared to men, which aligns with several previous studies [22,23,99], who also reported heightened emotional responses in women, especially in response to fear and sadness. However, this finding contrasts with some research, such as the work by [62], which reported minimal gender differences in discrete negative emotions. The discrepancy between our results and those of other studies might be attributable to cultural differences, sample characteristics, or methodological variations in emotion elicitation and measurement. Furthermore, our study found no significant gender differences in emotional valence, suggesting that while women may experience certain emotions more intensely, the overall positivity or negativity of these emotions is not markedly different between men and women. This aligns with previous research by [22,24,62,86], who found similar patterns in emotional valence across genders. This pattern of findings highlights the complexity of gender differences in emotional responses and underscores the need for further research to explore these dynamics in diverse cultural and demographic contexts.

Another significant contribution of our study was the introduction of four novel video clips specifically designed to elicit happiness. These clips were carefully selected and rigorously tested to ensure they met the criteria for evoking a strong positive emotional response, outperforming traditional stimuli such as those used by [22,23] (note that in one of our early pilots, we tested these earlier clips within an Iranian context, but they failed to elicit the desired level of happiness, leading us to develop our own). Unlike the often-used but less ecologically valid happy stimuli, our video clips featured dynamic and relatable human interactions that resonate more naturally with viewers. This not only resulted in more consistent happiness ratings but also avoided the potential confounds of other emotional stimuli that may inadvertently induce mixed emotions. While our happiness clips were not directly compared with other established happiness stimuli from databases such as those by [22,23,29], they demonstrated superior ecological validity and compatibility with other emotional video clips used in this and similar studies. Future research should aim to further validate these clips across different cultural contexts to establish their robustness and generalizability. Nonetheless, the successful implementation of these happiness-inducing clips marks a valuable advancement in the toolkit available for emotion research.

A further noteworthy contribution of this study is its in-depth examination of mixed feelings, which refers to the simultaneous experience of contrasting emotions, such as happiness and sadness. Our findings demonstrate that several video clips, initially categorized under a single emotion, also induced significant mixed feelings in participants, particularly those aligned with complex emotional experiences like nostalgia or bittersweetness. This finding is critical as it underscores the complexity of human emotional responses, challenging the discrete emotions framework that has dominated emotion research for decades. From a theoretical perspective, such co-activation is compatible with contemporary cognitive-affective accounts that treat emotional experience as graded and multi-component rather than strictly categorical, such that a single episode can recruit partially overlapping affective and appraisal-related components (e.g., high arousal together with ambivalent valence) [96,152–154]. In this sense, mixed feelings are not merely noise in validation, but an informative signature of complex affective episodes that normative databases should quantify and report [155,156]. Previous studies have either overlooked mixed feelings or treated them as anomalies (e.g., [94,95]), yet our results align with more recent research (e.g., [23,155,156]) that advocates for the inclusion of mixed emotions as a core component of emotional experience. The alignment of our findings with these contemporary studies emphasizes the necessity for emotion-eliciting tools to accommodate and accurately measure such complex emotional states. However, it is noteworthy that some clips failed to evoke the intended mixed feelings in the Iranian context, possibly due to cultural differences in emotional expression, a factor that has been underexplored in prior research (e.g., [157]). This highlights the need for continued refinement of emotion-eliciting databases to ensure their cross-cultural applicability.

A detailed comparison of our study with that of [26] reveals several critical distinctions and methodological advantages. One of the primary strengths of their work lies in its incorporation of neuropsychological measures, which provide valuable insights into the neural correlates of emotional processing. However, their study’s limited sample size poses constraints on the generalizability of their findings. In contrast, our study, with its larger sample size of 300 participants, offers a more robust and generalizable dataset, which is particularly important in the context of validation studies. Another critical difference is in the cultural representation within the study samples. While their research did not fully account for the cultural diversity within Iran, our study specifically aimed to include participants from various ethnic backgrounds, reflecting the multicultural nature of Iranian society. This approach ensures that our findings are more representative and applicable across the broader Iranian population. Additionally, while their study utilized locally produced Iranian films, we selected internationally recognized films with Persian subtitles. This choice was motivated by concerns that Iranian films might not effectively evoke basic emotions due to cultural and religious constraints, as commonly agreed upon by the Iranian population. Our hypothesis is that basic emotions are universally experienced and can be reliably induced using culturally neutral stimuli, as supported by prior research (e.g., [23]). Hence, using international films with appropriate Persian subtitles ensures that the emotional content is accurately conveyed, thereby maximizing the effectiveness of emotion induction. Moreover, our study introduces the exploration of “tenderness” as a discrete emotion, an aspect that was not covered in [26]. The inclusion of this emotion aligns with emerging trends in emotion research, which recognize the importance of a broader range of emotional experiences. Furthermore, we employed both the SAM and DES questionnaires to assess emotional responses, offering a comprehensive evaluation that includes both dimensional and discrete emotional measures. In contrast, [26] relied on the PANAS questionnaire, which has been critiqued in the literature for its ambiguity potentials due to overlapping variables (see [23]). Our study also took an additional step by validating the DES questionnaire for the first time within the Iranian community, contributing a valuable tool for future research in this field. Additionally, we examined mixed emotions and gender differences with a level of detail not fully addressed in the previous study, providing valuable insights into the complexity of emotional experiences. Lastly, while [26] collected data via a web-based approach, our study utilized a controlled laboratory environment, which allowed for greater control over external variables and thus increased the internal validity of our findings. In conclusion, while both studies make significant contributions to the field of emotion research, our work is distinguished by its larger and more representative sample, methodological comprehensiveness, and the introduction of novel emotional dimensions and validated tools.

Another contribution of this study is the development and validation of the DES in Persian, specifically within the Iranian community. To our knowledge, this is the first time that the DES has been adapted and rigorously tested for reliability and validity in this cultural context. S1 Appendix provide detailed insights into this process, where we conducted extensive statistical analyses, including reliability analysis, principal component analysis (PCA), and exploratory factor analysis (EFA). These analyses confirmed the DES as a robust tool for measuring emotional experiences in Iran, with a Cronbach’s alpha of 0.88 indicating strong internal consistency. The successful adaptation of the DES not only enhances the methodological rigor of emotion research within Iran but also offers a validated instrument for future studies exploring emotional dynamics in Persian-speaking populations.

While our study offers valuable insights into emotional responses elicited by video clips, several limitations should be acknowledged. The reliance on self-report measures in this study is a limitation that should be addressed in future research. While self-reports provide valuable insights into subjective emotional experiences, they may not fully capture the complexity of these experiences, particularly when it comes to the temporal dynamics of emotions [158,159]. Future studies should consider integrating psychophysiological indices (e.g., electrodermal activity and heart rate variability) and neurophysiological recordings (e.g., EEG/ERP), alongside neuroimaging and oculomotor methods (e.g., fMRI, MEG, and eye-tracking), to obtain a more comprehensive and multimodal characterization of emotional responses and to strengthen convergent validity beyond self-report [159,160]. Moreover, the participant sample was relatively homogeneous, consisting primarily of young adults from a specific cultural background. This limits the generalizability of our findings to more diverse populations, including different age groups and cultural backgrounds. Future research should aim to include a more representative sample to validate the findings across a broader demographic. Additionally, the use of video clips as stimuli, while effective for eliciting emotional responses, may not fully capture the range and complexity of emotions experienced in real-world scenarios. The standardized nature of these clips, while advantageous for experimental control, could affect the ecological validity of our results. Further research could explore the duration of emotional states induced by film clips, examining how long these emotions persist and what factors influence their retention or decay [145,161]. This line of inquiry is particularly relevant to distinguishing between short-lived emotional reactions and more prolonged mood states, a distinction that is critical for understanding the nature of affective experiences [159,161]. In addition, although we applied an explicit cultural/religious compatibility criterion to screen both the visual content and dialogue of candidate excerpts and presented all clips with professionally reviewed Persian subtitles, most source materials were drawn from internationally produced (predominantly Western) films. Therefore, even when the elicited affective profiles are robust, the depicted contexts, social scripts, and emotion display norms may not fully mirror everyday Iranian cultural settings, potentially limiting cultural-ecological generalizability. Importantly, related work in Persian stimulus development has begun to juxtapose locally produced Persian clips with non-Persian sets (e.g., English/French clips) primarily at the level of dimensional affect (valence/arousal) within a physiological-signal database [26]. Building on this direction, future work should more directly quantify stimulus-origin effects by systematically comparing locally produced Iranian clips against international clips within the same validation protocol, thereby isolating the contribution of culturally indigenous contexts beyond language adaptation alone. Another limitation is the lack of consideration for individual differences in emotional processing, such as personality traits, previous emotional experiences, or current mood states, which could significantly impact how participants respond to the film clips. Future studies could benefit from assessing these variables to better understand their influence on emotional responses. In addition, because all raw ratings and the derived clip-level measurement matrix are openly shared, future work can leverage these data as a benchmark for supervised machine learning/deep learning models that predict emotion categories (and mixed-emotion profiles), and can extend such predictive modeling by combining self-report with multimodal physiological recordings for automated emotion recognition. Finally, the cross-sectional design of this study, which captures emotional responses at a single point in time, may not reflect the dynamic nature of emotional experiences. Longitudinal studies could provide more comprehensive insights into how emotional responses evolve over time or with repeated exposure, offering a deeper understanding of the stability and variability of these responses across different contexts and over extended periods.

The establishment and validation of this database represent a significant contribution to the field of emotion research, particularly within the context of Iranian society. By including a diverse sample and considering gender differences, and creating a culturally sensitive tool that accommodates the linguistic and cultural diversity of Iran, this study offers a valuable resource for researchers interested in the cultural dimensions of emotion. The database is made accessible online, providing researchers with the opportunity to further validate and expand upon this work, thus contributing to a more comprehensive understanding of how emotions are experienced across different cultures.

## Conclusion

This study successfully developed a culturally sensitive tool for emotional research in Iran, addressing a critical need in the field. Future studies should continue to explore the cultural dimensions of emotion, utilizing a variety of methodological approaches to deepen our understanding of affective processes across different societies.

## Supporting information

S1 Appendix Reliability and validity of the persian translation of the differential emaotions scale (DES) in the Iranian comminity. (PDF)

S1 Cheklist Inclusivity in global research. (PDF)
