# Supplementary material for: Building bridges to emotion: Developing a standardized film-based emotion elicitation tool for Iranian culture
Source: PLoS One. 2026 Mar 12;21(3):e0343598. doi: 10.1371/journal.pone.0343598 (PMC12981497; doi:10.1371/journal.pone.0343598)
Supplement: S1 Appendix — (PDF) [file pone.0343598.s001.pdf]

Building bridges to emotion: developing a standardized film-based emotion elicitation tool for Iranian culture

Milad Yousefi
Institute for Cognitive and Brain Sciences, Shahid Beheshti University, Tehran, Iran
Jamal Amani Rad*
Choice Modelling Centre, University of Leeds, Leeds, UK

February 16, 2026

S1 Appendix.

Reliability and validity of the persian translation of the differential emotions scale (DES) in the Iranian community

The Persian translation of DES has been developed as part of this study. To evaluate the validity and reliability of this version of DES in the Iranian community, where 300 participants were exposed to 27 emotional video clips, we conducted a comprehensive analysis involving several statistical techniques, such as reliability analysis, principal component analysis (PCA), and exploratory factor analysis (EFA). These techniques were chosen to ensure that the DES is a reliable and valid measure of emotional experiences in this specific experimental setting. The primary focus was on responses to 16 items of the DES, which were analyzed for internal consistency and factor structure.

Internal Consistency and Reliability

To begin assessing the reliability of the DES, we first examined the inter-item correlations using a heatmap. This visual tool provided an initial overview of how each item on the DES correlated with the others, offering insights into the relationships between the items. The heatmap (see Fig S1) revealed that certain items exhibited strong correlations, while others were more weakly related. This pattern suggested that the items were likely measuring related but distinct aspects of emotional experiences, setting the stage for further reliability and factor analysis. Next, we calculated Cronbach's alpha to determine the internal consistency of the DES. This analysis is crucial because it helps to determine whether the set of items on the DES are reliably measuring the same underlying construct—emotional experiences. The reliability analysis yielded a Cronbach's alpha of 0.88 (with an average standard error, ASE, of 0.0038), indicating strong internal consistency (see Fig S2). This suggests that the items are well-correlated and reliably measure the underlying construct of emotions. The average inter-item correlation was 0.32, within the expected range for psychological scales, suggesting that the items are sufficiently correlated without being redundant. This high level of internal consistency justified proceeding to more detailed factor analyses. Additionally, the scale's reliability remained high when individual items were removed, with Cronbach's alpha values ranging from 0.87 to 0.89, and the 95% confidence intervals consistently indicating high reliability. The strong internal consistency observed here provided the necessary foundation to proceed with more detailed analyses, such as factor analysis, to explore the underlying dimensions of emotional experiences measured by the DES.

*Correspond: Email: j.amanirad@leeds.ac.uk; j.amanirad@gmail.com (JAR)

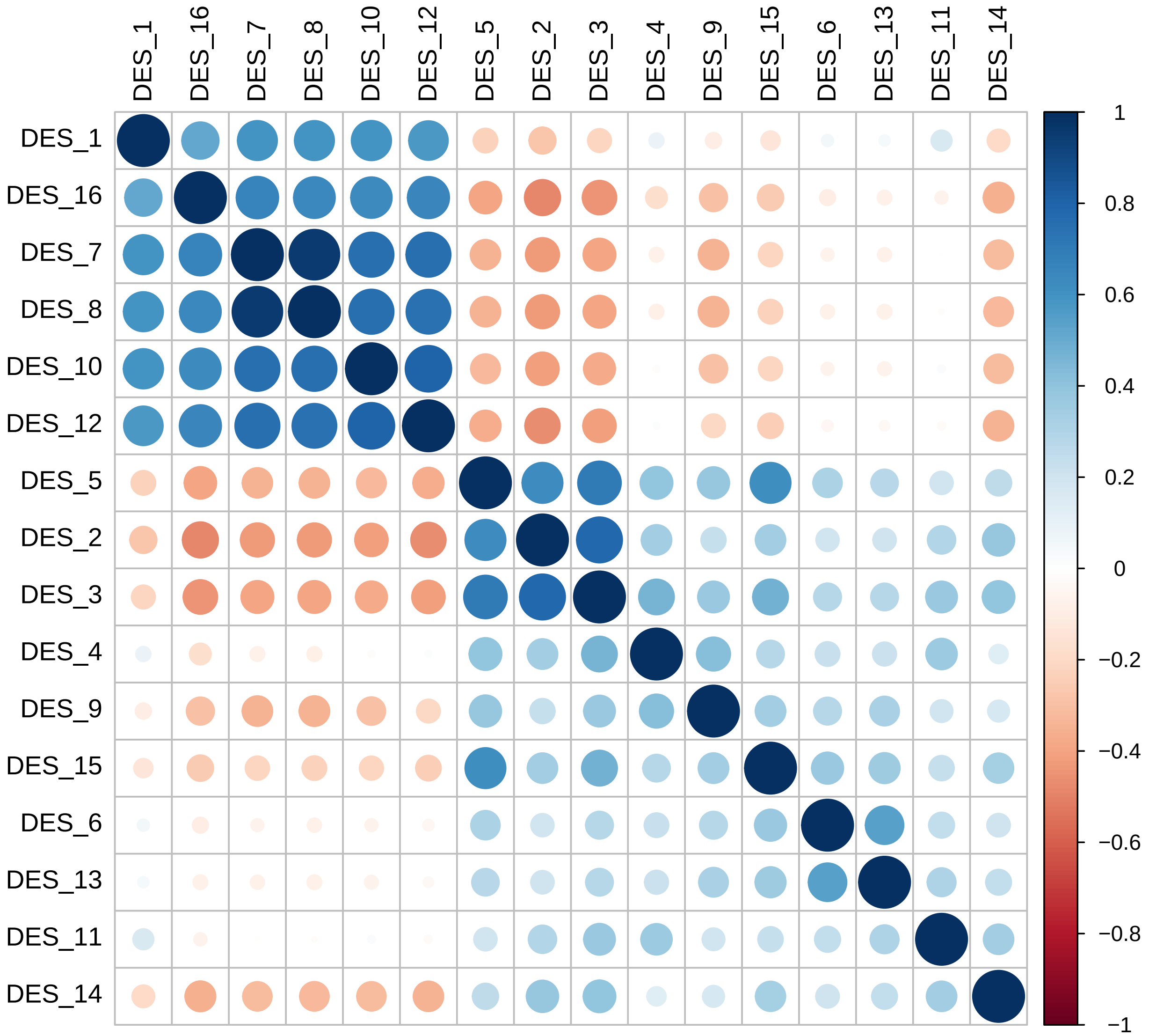

Fig S1. **Correlation matrix of DES items from 1 to 16.** The plot displays the correlations between different DES items, with color intensity indicating the strength of the correlation.

Exploratory Factor Analysis (EFA) Preparation: KMO and Bartlett's Test

Before performing factor analysis, we assessed the suitability of our data for this type of analysis by conducting the Kaiser-Meyer-Olkin (KMO) Measure of Sampling Adequacy and Bartlett's Test of Sphericity. The KMO value was higher than 0.85 (see Fig S3), which is well above the commonly recommended threshold of 0.6, indicating that the sample size and the item correlations are adequate for factor analysis. Also, Bartlett's Test of Sphericity was significant ($p < 0.00001$), confirming that the correlation matrix is not an identity matrix and that there are sufficient correlations among variables to justify the use of factor analysis. These preliminary checks are needed as they provide the statistical foundation for the subsequent factor analyses, ensuring that the data are appropriate for extracting meaningful factors.

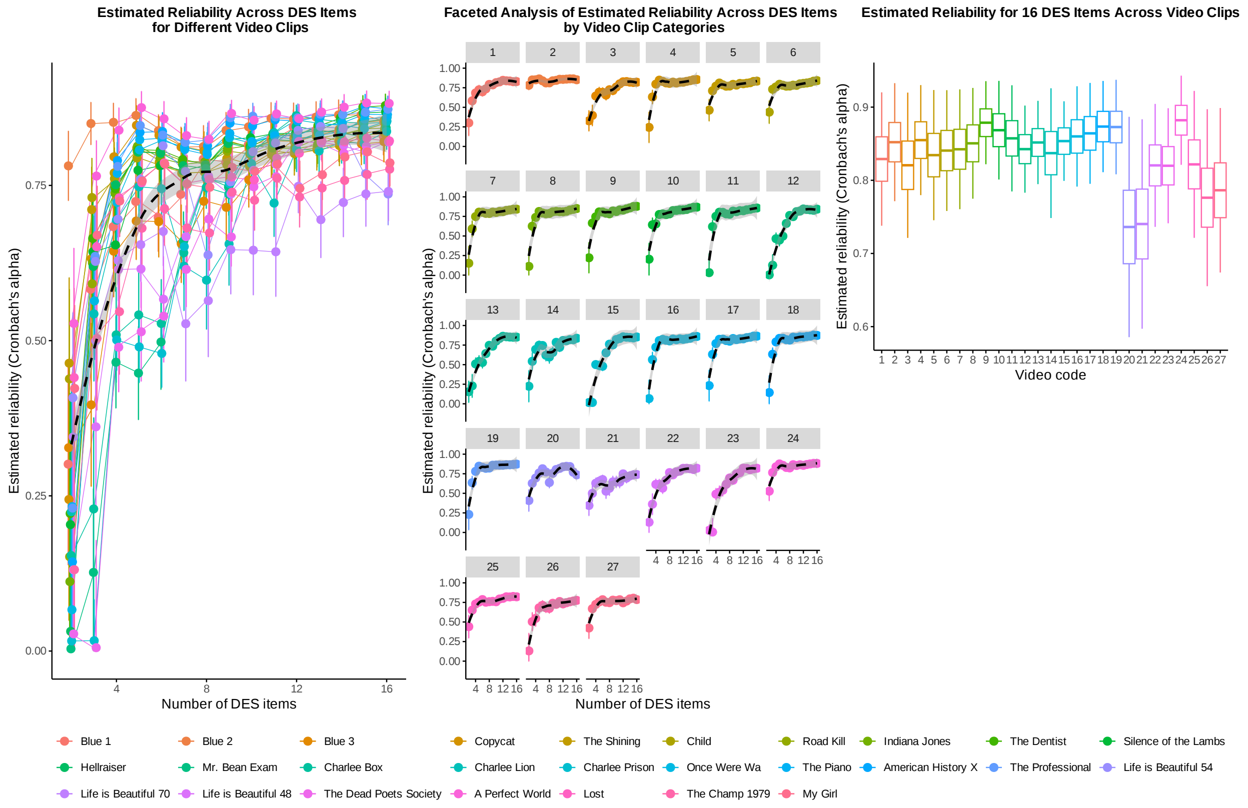

Fig S2. **Combined visualizations of estimated reliability (Cronbach's alpha) across DES items and video clips.** The plot in the top right displays the relationship between the number of DES items and estimated reliability, with lines, error bars, and smoothed trends for different video clips. The left plot shows a faceted analysis of estimated reliability across DES items, organized by video clip categories, highlighting variations within each category. The bottom right plot presents a boxplot of estimated reliability for 16 DES items across various video clips, illustrating the distribution of reliability estimates by video code.

Principal Component Analysis (PCA)

To explore the underlying structure of the DES, we conducted a Principal Component Analysis (PCA). PCA is a dimensionality reduction technique that helps to identify the principal components (factors) that explain the maximum variance in the data. Fig S4 revealed a clear structure with two significant components, suggesting that the DES items primarily load onto two factors. These components account for the majority of the variance in the data, indicating that the DES items are likely measuring two distinct aspects of emotional experiences. This initial PCA provided a preliminary understanding of the data's dimensionality, which was further explored in subsequent analyses.

Exploratory Factor Analysis (EFA)

To better understand the factor structure suggested by the PCA, we conducted an Exploratory Factor Analysis (EFA) with a varimax rotation, which is commonly used to achieve a simpler, more interpretable factor structure. The EFA results indicated a two-factor solution, aligning with the PCA findings. The rotated factor loadings showed that the items clustered into two distinct factors, which we hypothesize correspond to different dimensions of emotional experiences—potentially ”arousal” and ”valence” (or perhaps ”positive affect” and ”negative affect”), though further interpretation is necessary to precisely define these factors. The clear loading of items onto these two factors supports the theoretical construct of the DES. This provided preliminary evidence that the DES is a valid instrument for assessing emotional experiences

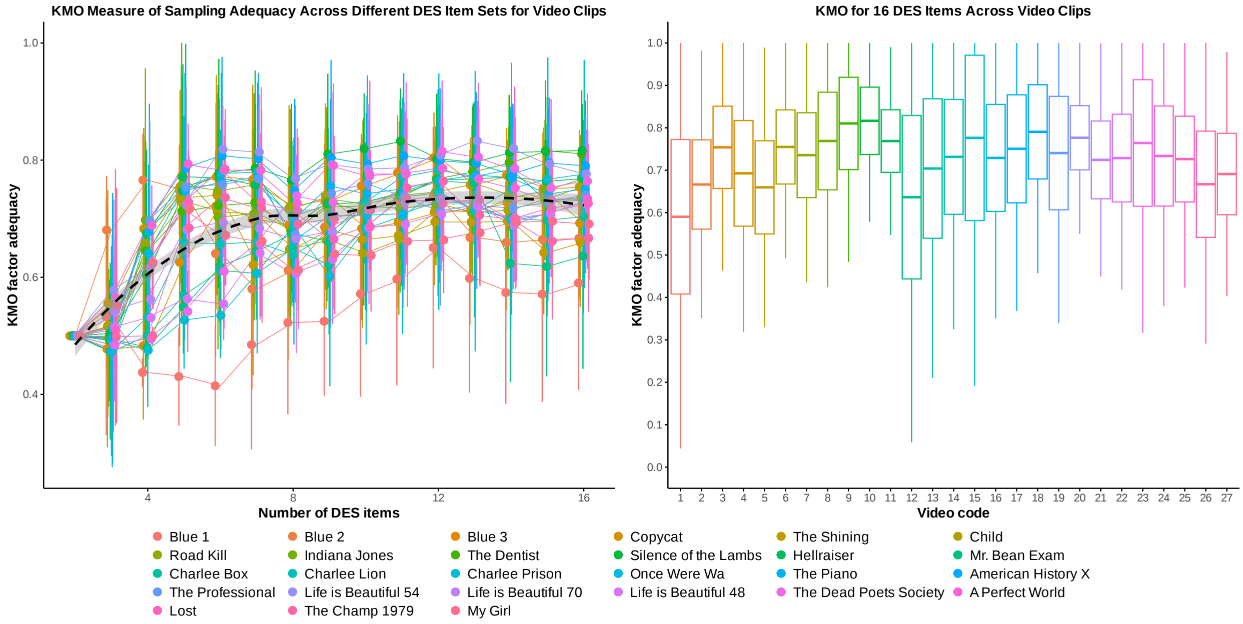

Fig S3. **KMO Measure of Sampling Adequacy**. Left: KMO scores across different numbers of DES items for various video clips, with lines and error bars. Right: Boxplot of KMO scores for 16 DES items by video clip, showing variability in factor adequacy.

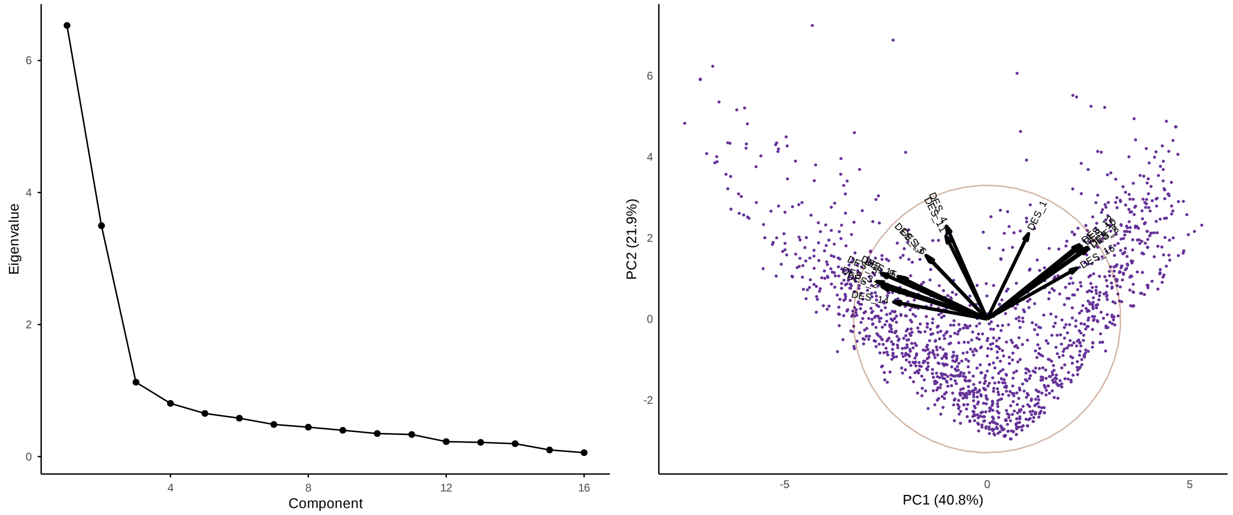

Fig S4. **Principal Component Analysis Results.** (Left) Plot of Eigenvalues: This plot displays the eigenvalues of the principal components, showing the variance explained by each component and helping to determine the number of significant components. (Right) PCA Biplot: This biplot visualizes the principal components and their associated variable vectors. The plot illustrates the relationships between variables and the distribution of observations in the PCA space.

in the context of induced emotional states through film clips.

The analyses conducted here provide strong evidence for the reliability and validity of the Persian trans-

lation of DES in the context of mood induction through video clips in the Iranian community. The initial correlation matrix and heatmap offered insights into the relationships between items, setting the foundation for more complex analyses. The high internal consistency, coupled with the robust factor structure identified through PCA and EFA, supports the use of DES in experimental settings involving mood induction.

These findings underscore the DES's utility in experimental settings, particularly in research involving emotional manipulation in Iranian culture. The validated factor structure enhances our understanding of how different dimensions of emotional experiences are measured and triggered by emotional stimuli. Overall, the Persian translation of DES proves to be a reliable and valid tool for assessing emotions, offering valuable insights for future research in this field.

Finally, the Persian translation of DES is provided below, with English items shown side-by-side for transparency and ease of comparison. For comprehensive details on the S1 Appendix, including the translation and data, please refer to the full documentation available at https://osf.io/2td5n/.

Differential Emotions Scale (DES) پرسشنامه

پرسشنامه زیر دارای 16 سوال می باشد، که هر سوال به گروهی از صفات هیجانی تعلق دارد. شما باید بر اساس اینکه این گروه کلمات تا چه اندازه وصف کننده وضعیت هیجانی شما بر اساس فیلم مشاهده شده هستند یک نمره از **1 (نه اصلا)** **الی 7 (خیلی شدید)** بدهید.

شما برای هر سوال فقط می توانید یک گزینه از 1 الی 7 انتخاب کنید.

7	6	5	4	3	2	1			
خیلی شدید	نسبتا زیاد	زیاد	متوسط	کم	خیلی کم	نه اصلا			
							علاقمند، متمرکز، هشیار	interested, concentrated, alert	**1**
							هراسناک وحشت زده، ترسیده	fearful, scared, afraid	**2**
							مضطرب، تنیده، عصبی	anxious, tense, nervous	**3**
							متاثر	moved	**4**
							خشمگین، برافروخته ، عصبانی	angry, irritated, mad	**5**
							شرمنده، خجالت زده	ashamed, embarrassed	**6**
							خونگرم، شاد، سرحال	warm hearted, gleeful, elated	**7**
							سرخوش، سرگرم ، خوشحال	joyful, amused, happy	**8**
							غمگین، دلسرد، اندوهگین	sad, downhearted, blue	**9**
							راضی، خشنود	satisfied, pleased	**10**
							متعجب، شگفت زده، بهت زده	surprised, amazed, astonished	**11**
							دوست داشتنی، با محبت، صمیمی	loving, affectionate, friendly	**12**
							احساس گناه، پشیمان	guilty, remorseful	**13**
							منزجر، حال گیر، بیزار	disgusted, turned off, repulsed	**14**
							اهانت کننده، تحقیرکننده، گستاخ	disdainful, scornful, contemptuous	**15**
							راحت، متین، آرام	calm, serene, relaxed	**16**
